# Morphine-induced changes in the function of microglia and macrophages after acute spinal cord injury

**DOI:** 10.1186/s12868-022-00739-3

**Published:** 2022-10-10

**Authors:** Mabel N. Terminel, Carla Bassil, Josephina Rau, Amanda Trevino, Cristina Ruiz, Robert Alaniz, Michelle A. Hook

**Affiliations:** Department of Neuroscience and Experimental Therapeutics, Texas A&M Health Science Center, 8447 Riverside Parkway 47, Bryan, TX 77807 USA

**Keywords:** Spinal cord injury, Inflammation, Opioids, Morphine, Microglia, Macrophages

## Abstract

**Background:**

Opioids are among the most effective and commonly prescribed analgesics for the treatment of acute pain after spinal cord injury (SCI). However, morphine administration in the early phase of SCI undermines locomotor recovery, increases cell death, and decreases overall health in a rodent contusion model. Based on our previous studies we hypothesize that morphine acts on classic opioid receptors to alter the immune response. Indeed, we found that a single dose of intrathecal morphine increases the expression of activated microglia and macrophages at the injury site. Whether similar effects of morphine would be seen with repeated intravenous administration, more closely simulating clinical treatment, is not known.

**Methods:**

To address this, we used flow cytometry to examine changes in the temporal expression of microglia and macrophages after SCI and intravenous morphine. Next, we explored whether morphine changed the function of these cells through the engagement of cell-signaling pathways linked to neurotoxicity using Western blot analysis.

**Results:**

Our flow cytometry studies showed that 3 consecutive days of morphine administration after an SCI significantly increased the number of microglia and macrophages around the lesion. Using Western blot analysis, we also found that repeated administration of morphine increases β-arrestin, ERK-1 and dynorphin (an endogenous kappa opioid receptor agonist) production by microglia and macrophages.

**Conclusions:**

These results suggest that morphine administered immediately after an SCI changes the innate immune response by increasing the number of immune cells and altering neuropeptide synthesis by these cells.

**Supplementary Information:**

The online version contains supplementary material available at 10.1186/s12868-022-00739-3.

## Background

Pain is frequently experienced within the first few hours following spinal cord injury (SCI). Moreover, two-thirds of the people living with SCI continue to experience pain for months and even years after the injury, and about one-third rate their pain as severe or neuropathic [1, 2]. There is evidence to suggest that effective treatment of acute pain may prevent the chronification of pain [3–5]. Unfortunately, however, our previous data suggest that the standard use of opioids for treating pain in the emergency setting can negatively affect recovery after SCI.

In the clinical population, we have found that high doses of opioids administered in the first 24 h post SCI are associated with increased symptoms of chronic pain at 1-year post injury [6]. Our data from a rat SCI model suggests that opioid administration *causes acute and long-term pain*, rather than a correlative association with higher pain leading to greater opioid use. In the rat model, our laboratory has repeatedly found that morphine given in the days following the injury significantly undermines locomotor function, increases cell death around the lesion, and increases chronic pain [7–15]. Given the limited number of effective analgesics, it is critical that we develop a better understanding of the molecular consequences of activating opioid receptors within the highly inflammatory context of SCI.

We hypothesize that morphine activates opioid receptors (ORs) on microglia and macrophages and changes their expression and function, fundamentally altering the innate immune response to SCI. While immune cell activation immediately after SCI prevents injury damage from expanding indiscriminately along the cord [16], treatments that protract or augment the initial inflammatory response can delay injury resolution [17]. We have previously reported an increase in inflammatory cells at the injury site after a single intrathecal (i.t.) dose of morphine in SCI rats [15]. We have also found that pre-treatment with the glia-inhibitor, minocycline, protects against the negative effects of a single dose of morphine [15], implicating glial activation in the adverse effects of the analgesic. These data suggest that an opioid-immune interaction could be at the core of the negative effects that we observe in motor recovery after morphine administration.

It is well known that opioids can modulate microglia and macrophages by activating surface receptors, thus establishing a plausible mechanism through which morphine can affect the development of the inflammatory response. For example, opioid receptor activation can promote the proliferation of microglia and macrophages [18]. We have also found that morphine after SCI increases the expression of the inflammatory cytokine IL-1β [9]. A shift in the phenotypic profile of immune cells could have significant implications for the production of inflammatory cytokines and the overall neurotoxic environment at the site of injury.

Morphine can also bind to classic opioid receptors [19, 20] to initiate context-dependent molecular cascades that can induce neurotoxicity [21–23], including the p38 MAPK pathway [22, 24, 25] and the ERK 1/2 pathway which is responsible for CREB regulation of dynorphin gene expression [26]. Dynorphin, the endogenous KOR agonist, can induce neurotoxicity at elevated levels, and even causes motor dysfunction in uninjured rats when administered intrathecally [27]. Interestingly, pre-treatment with Norbinaltorphimine (nor-BNI), a kappa opioid receptor (KOR) antagonist, also prevents the negative effects of intrathecal morphine on motor recovery after SCI [13]. However, the extent to which intravenous (i.v.) morphine administration might alter these key functions and delay injury resolution is not known.

To address this, in the present study, we used flow cytometry to characterize the changes in expression and phenotype of CD11b^+^CD45^low^ (microglia) and CD11b^+^CD45^high^ (infiltrating macrophages) cells after SCI and i.v. morphine administration. Notably, while we refer to CD11b^+^CD45^+^ cells as microglia and macrophages, as is convention, it is acknowledged that CD11b^+^CD45^+^ cell populations might also include other immune cell populations including lymphocytes, natural killer cells, and neutrophils, depending on the timepoint examined. Future studies should include markers for these cell types. Next, we used Western blot analysis to investigate whether morphine changes the function of CD11b^+^ cells by altering the expression of key proteins within inflammatory and apoptotic signaling pathways. We found that morphine increases the number of microglia and macrophages at the injury site, and increases their production of β-arrestin, ERK 1/2, p38, dynorphin, and pro-dynorphin. Taken together, our results suggest that administration of morphine within the highly-inflammatory context of SCI changes the number and function of key immune cells. The shift in expression and phenotype in the overall immune response to the injury is likely one of the contributing mechanisms through which morphine delays injury resolution and decreases recovery of function after SCI.

## Methods

### Subjects

A total of 192 Male Sprague Dawley rats obtained from Envigo (Houston, TX), between 90 and 110 days old (300–350 g), served as subjects. Eighty rats were used in the flow cytometry study, and 112 rats were used to collect protein for Western blot analysis. In addition to the 192 animas included in these analyses, 16 rats either died or were removed from the study based on a priori excussion parameters, such as rats that had a BBB score higher than 4 at day 1 post-injury assessment (see assessment of locomotor activity). This study focused on males only as there is a gender bias observed in the clinical setting with the incidence of SCI being about 3 times higher for males than females [28, 29]. Rats were single-housed in plexiglass cages (45.7 (length) × 23.5 (width) × 20.3 (height) cm) with food and water available ad libitum. Following the contusion (or sham) surgery, body weight was recorded daily. Rats that received the contusion surgery also lost bladder function, and they were manually expressed daily in the morning (7:00–9:30) and evening (16:30–18:00) until an empty bladder was observed for 3 consecutive days [8, 12, 13]. Rats were maintained on a 12-h light/dark cycle, with behavioral testing conducted during the light cycle.

### Jugular catheter surgery

A jugular catheter was implanted 5 days prior to the contusion injury for intravenous drug delivery. For the surgery, anesthesia was induced with isoflurane at a level of 5% gas and lowered to 2–3% to maintain a stable level of anesthesia for the duration of the surgery. The subject’s back and neck area were shaved and disinfected with iodine and ethanol. A horizontal incision (4 cm) was then made on the back of the subject below the shoulders. Placing the rat on its right side, a subcutaneous tunnel beginning at the large opening in the back, traveling over the shoulder, and ending at the neck was made by carefully separating the skin from the muscle using forceps. Next, a superficial incision (1 cm) was made directly above the jugular vein and connective tissue was carefully cleared away from the vein to allow for insertion of the catheter. A sterilized catheter consisting of Silastic tubing (0.025-mm ID) was inserted through the incision in the back and passed subcutaneously over the right shoulder to reach the opening at the neck. Before insertion, the catheter was flushed with sterile saline by attaching a syringe to the catheter’s cannula; the syringe remained attached to the catheter for the duration of the surgery. The Silastic tubing was inserted into the vein using an 18-G needle as a guide and kept in place by tying loose knots to both ends of the jugular vein. Successful placement of the i.v. line was confirmed by pulling on the syringe attached to the catheter to draw back blood. The back-mount pedestal (model 313-00BM-10-SPC; Plastics One Inc., Roanoke, VA) connected to the catheter was implanted subcutaneously and the tip of the catheter’s cannula was allowed to exit the skin through a small opening at the back between the scapulae. All wounds were closed using Michel clips. After the surgery, the catheter was flushed with heparin-saline (1000 units/mL) and immediately capped to prevent the formation of blood clots inside the tubing. For the first 24 h after surgery, rats were placed in a recovery room maintained at 26.6 °C. To compensate for fluid loss, rats were given 3 mL of saline after surgery [10].

### Contusion surgery

Rats received a moderate contusion injury using the Infinite Horizon (IH) spinal cord impactor (PSI, Fairfax Station, VA, USA). As described in Brakel, Aceves [30] rats were anesthetized with isoflurane (5%, gas), and after a stable level of anesthesia was reached, the concentration of isoflurane was lowered to 2–3%. The subject’s back was shaved and disinfected with iodine and a 5.0 cm incision was made over the spinal cord. Two incisions were made along the vertebral column, on each side of the dorsal spinous processes, extending about 2 cm rostral and caudal to the T12 segment. Musculature and connective tissue around the transverse processes was cleared to allow for clamping of the vertebral spinal column. Next, the dorsal spinous process at T12 was removed (laminectomy), and the spinal tissue was exposed. The dura remained intact. The vertebral column was fixed within the IH device using two pairs of Adson forceps. A moderate injury was produced using an impact force of 150 k dynes and a 1 s dwell time on the exposed spinal tissue (vertebral level T12). The wound was closed using Michel clips. Sham surgery was conducted in the same way as the contusion, but without impacting the spinal tissue to damage to the nerve fibers. For the first 24 h after surgery, rats were placed in a recovery room maintained at 26.6 °C. To compensate for fluid loss, rats were given 3 mL of saline after surgery.

### Assessment of sensory reactivity

#### Tactile reactivity

Mechanical reactivity was tested by applying von Frey filaments (Semmes–Weinstein Anesthesiometer, Stoelting Co., Chicago, IL, USA) to the plantar surface of the hindpaws, as previously described [31, 32]. The test was conducted immediately after assessing locomotor behavior (using the BBB scale rating). The tactile reactivity test was performed twice on each of the testing days, once immediately before starting drug administration and then again 30-min after the last dose was given. Filaments of increasing diameter (indicative of force applied) were briefly pressed against the rat’s paw until they exhibited a motor (hindpaw withdrawal) and vocal response. The intensity of the stimuli that produced a response was reported using the formula provided by Semmes–Weinstein: Intensity = log10 (10,000 * G force). If one or both responses (motor and vocal) were not observed, testing was terminated at a force of 300*g*. Tests of mechanical and thermal reactivity were conducted immediately before and 30 min after treatment on days 1 and 7 of drug administration.

#### Thermal reactivity

To assess acute drug efficacy, the tail-flick test [32–34] of thermal reactivity was conducted immediately after assessing tactile reactivity. The tail-flick test was performed twice per day on Days 1 and 7 post injury, once before starting drug administration and again 30-min after the last dose was given. The methods for testing tail-flick reactivity have been described in our previous papers [8, 12, 15]. Briefly, rats were placed in restraining tubes and allowed to acclimate to the tail-flick apparatus (IITC Life Science Inc., Woodland Hills, CA, USA) and testing room (maintained at 26.5 °C) for 10 min. Prior to testing, the temperature of the light, focused on the tail, was set to elicit a baseline tail-flick response in approximately 4 s in an intact rat. This pre-set temperature was maintained across all SCI rats. In testing, the latency to flick the tail away from the radiant heat source (light) was recorded. If a subject failed to respond, the test trial was automatically terminated after 8 s of heat exposure. Two tests occurred at 2 min intervals, and the last tail-flick latency time was recorded.

### Assessment of locomotor recovery

Locomotor behavior was assessed once a day for the first 7 days post injury immediately prior to daily drug administration, using the Basso, Beattie and Bresnahan (BBB) scale [35] in an open enclosure (a blue children’s wading pool, 99 cm in diameter, 23 cm deep). The BBB scale is a 21 point scale that assesses movement of the hip, knee and ankle joints, as well as plantar weight-supported stepping and coordination between the limbs. Higher scores reflect greater recovery of locomotor function. For example, a score of 4 indicates slight movement of all 3 joints, a score of 10 denotes extensive movement of all joints with occasional weight-supported plantar stepping, and a score of 14 indicates consistent stepping with consistent coordination between the limbs and some deficits in fine motor movement (i.e. paw rotation at landing or lift off). For our analyses, locomotor scores using the original BBB scale were transformed, as described in previous publications [36], to ensure data were amendable to parametric analyses. In short, this transformation removed a discontinuity in the BBB scale and made data suitable for parametric statistics. With the transformed scores, higher scores still indicate higher function, however the subjects can only reach a maximum score of 12. With a moderate contusion injury, and particularly with morphine treatment, fine motor movements are rarely recovered.

Baseline motor function was assessed on the day following injury and prior to drug treatment. As described in our previous studies [7, 12, 14], the rats were placed in the open field and observed for 4 min. Rats that had an average BBB score higher than 4 points on the day following contusion injury were excluded from the experiment. The remaining subjects were scored prior to daily drug treatment until euthanasia. Care was taken to ensure that all investigators’ scoring had high intra- and inter- observer reliability (all r’s > 0.89) and that they were blind to the subject’s experimental treatment.

### Intravenous drug administration

Drug administration began 24 h following the contusion injury, after performing baseline tests for locomotor function, thermal reactivity, and mechanical reactivity. Baseline BBB scores were balanced across groups. All drugs (morphine, saline, and heparin-saline) were administered via the i.v. catheter. For both the flow cytometry and Western blot experiments, half of the rats in each injury condition were treated with 10 mg of morphine (i.v.) on days 1–2, 20 mg on days 3–4, and 30 mg on days 5–7. Morphine was administered in 5 mg doses in 0.5 mL of saline at 1-h intervals up to the total daily dose. The remaining rats served as controls, receiving an equivalent volume of vehicle (0.9% sterile-saline) on the same schedule as the morphine-treated rats. Heparin-saline (1000 units/mL) was used to flush the catheters following drug administration. The doses outlined in the current study were derived from previous experiments assessing analgesia, self-administration, and cell expression after SCI [10, 14].

### Tissue collection and cell dissociation

Rats were euthanized on days 2, 4, or 8 (24 h after the final dose of morphine given on days 1, 3, or 7). For the Western blot analysis, morphine was administered for 3 days post injury and tissue was collected 30 min or 24 h after the final administration, to assess effects when the drug was active as well as long-term changes. The rats were deeply anesthetized (100 mg/kg of beuthanasia, i.p.) and perfused intracardially with 100 mL of cold 1X PBS. A 1.5 cm segment of the lesioned spinal cord was collected, or the equivalent area for sham rats. To prepare samples for both flow cytometry and Western blot analysis, the spinal tissue was mechanically dissociated using a blade and washed in 1X HBSS. Cells were retrieved by centrifugation (500×*g*, 5 min, 4 °C). The cell suspension was then enzymatically (Neural Tissue Dissociation Kit (P), Miltenyi Biotec, Germany) and mechanically dissociated using a dounce homogenizer with a loose pestle. The resulting cell suspension was washed twice with 1X HBSS and resuspended in 3 mL of 1X HBSS. The 3 mL suspension containing a heterogeneous mixture of dissociated cells (i.e. inflammatory cells, glia, and neurons) was filtered through a 70-micron cell strainer, washed twice, and counted (Countess; Life Technologies, Carlsbad, CA, USA) to assess cell viability. We established a successful strategy for removal of debris and dead cells by conducting a rigorous series of washes and centrifugation steps, as verified by two different live/dead staining methods (see Additional file 1).

### Flow cytometry

**Fig. 1:**
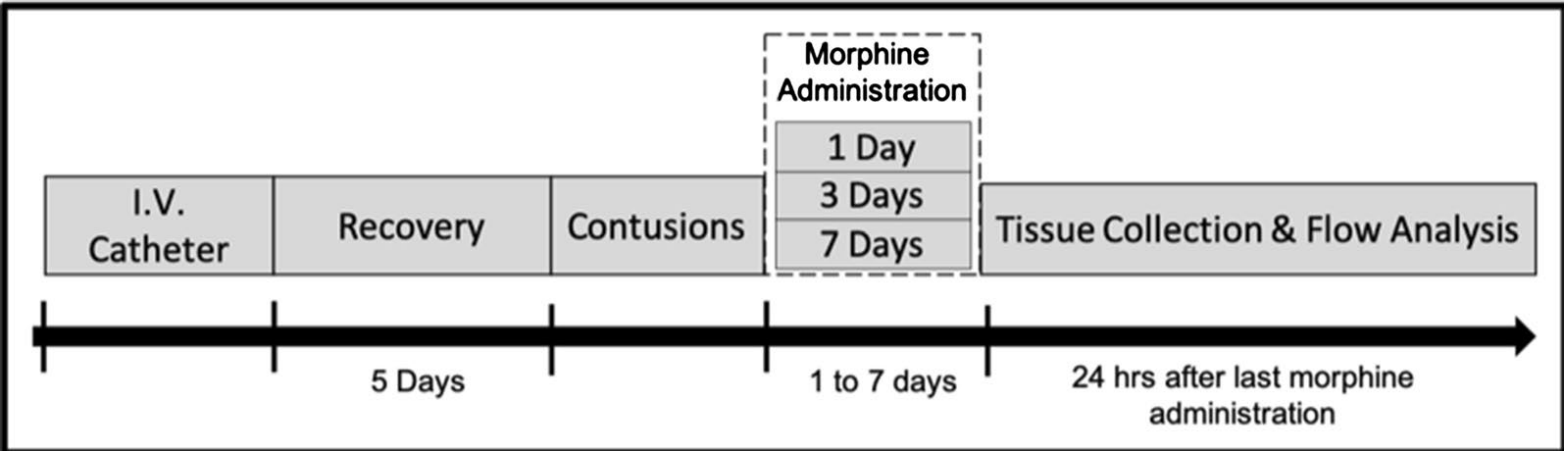
Timeline for flow cytometry experiments

Flow cytometry was used to quantify immune-competent cells at the lesion site (Fig. 1). From a single cell suspension of heterogeneous spinal cord cells, approximately 300,000 cells/well were plated in a round bottom 96-well plate and resuspended in 100 uL of staining buffer (BioLegend, San Diego, CA, USA) in preparation for immunostaining. To prevent spillover between fluorescent channels, we carefully tested different combinations of conjugated antibodies to design the M1 (CD86+) and M2 (CD206+) panels. The cells were then incubated with antibodies according to Table 1. For each subject, 2 technical replicates were prepared per set and phenotyped using a FACSFortessa flow cytometer (BD Biosciences, Franklin Lakes, NJ, USA). A total of 100,000 events per sample were recorded and data was analyzed using FlowJo software (Tree Star).

**Table 1:** List of antibodies used for flow cytometry

Antibody	Concentration	Manufacturer	Catalog No
*Set 1—CD86*+ *Pro-inflammatory phenotype*			
CD11b conjugated to biotin	1:1000	ThermoFisher, Waltham, MA, USA	MA5-17508
CD45 conjugated to eFluour450	1:100	ThermoFisher, Waltham, MA, USA	48-0461-82
Rabbit monoclonal to Kappa Opioid Receptor	1:500	Abcam, Cambridge, UK	ab183825
CD86 conjugated to FITC	1:100	ThermoFisher, Waltham, MA, USA	11-0860-82
Zombie NIR (Live/Dead Stain)	1:500	BioLegend, San Diego, CA, USA	423106
Native Streptavidin conjugated to PE/Texas Red	1:50	Abcam, Cambridge, UK	ab136195
Goat anti-rabbit Alexa 750	1:1000	ThermoFisher, Waltham, MA, USA	A-21039
*Set 2—CD206*+ *phagocytic phenotype*			
CD11b conjugated to FITC	1:1000	ThermoFisher, Waltham, MA, USA	MA5-17510
CD45 conjugated to eFluour450	1:100	ThermoFisher, Waltham, MA, USA	48-0461-82
Rabbit monoclonal to Kappa Opioid Receptor	1:500	Abcam, Cambridge, UK	ab183825
CD206 conjugated to biotin	1:50	Antibodies Online, Germany	ABIN181884
Zombie NIR (Live/Dead Stain)	1:500	BioLegend, San Diego, CA, USA	423106
Native Streptavidin conjugated to PE/Texas Red	1:50	Abcam, Cambridge, UK	ab136195
Goat anti-rabbit Alexa 750	1:1000	ThermoFisher, Waltham, MA, USA	A-21039

### Flow gating strategy

**Fig. 2:**
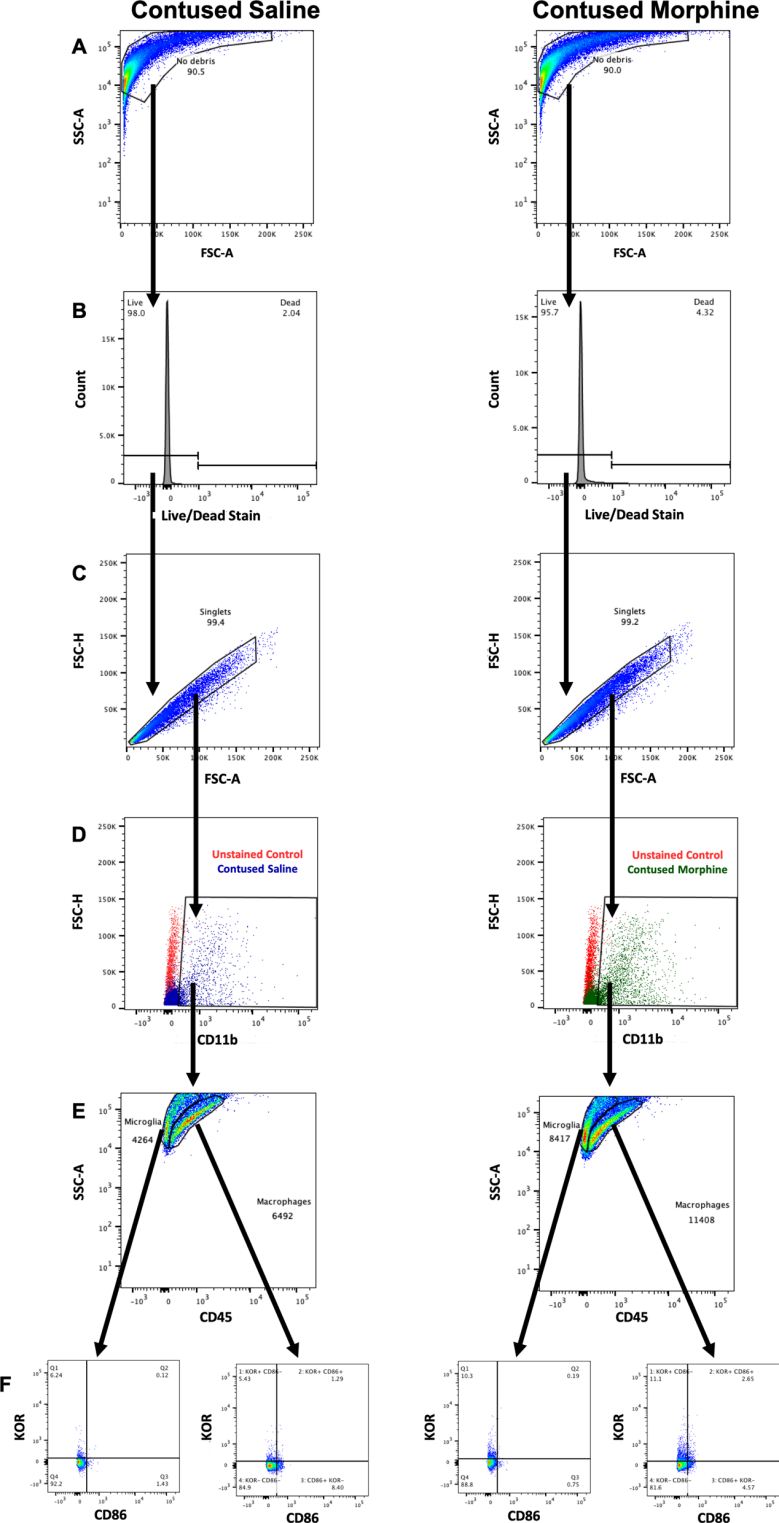
Flow cytometry gating strategy for CD86 set. Forward and side scatter were used to remove debris (**A**). Dead cells internalize the Zombie dye and give a positive signal, allowing for further exclusion (**B**). Singlets were then selected using height and area of forward scatter (**C**). Microglia and macrophages were identified by selecting cells that were positive for CD11b (**D**). CD45 was then used to differentiate between infiltrating macrophages (CD45high) and resident microglia (CD45low) (**E**). A quadrant system was created paring KOR vs. CD86 (M1 marker) into CD45-high and CD45-low cell populations (**F**)

In the flow cytometry experiments, the following gating strategy was used to identify cells of interest (see Fig. 2). First, cell debris was gated out using forward and side scatter. Then, viability was assessed using a zombie die (BioLegend, San Diego, CA). Dead cells internalize the fluorescent dye and give a positive signal, allowing for exclusion; all other cells were considered “live” cells. To control for cellular auto-fluorescence, unstained samples were prepared. To be considered positive (+), cell populations were selected using a gate that contained < 1% CD11b+ unstained cells. CD11b is expressed on a variety of immune cells, including neutrophils, monocytes, natural killer cells, and some lymphocytes [37–40]. Therefore, we quantified the total number of CD11b+ cells as an indirect measure of inflammation. Different CD11b antibodies were used for the CD86 and CD206 panels and the gating strategy was lightly adjusted to accommodate for differences in autofluorescence baseline in different channels.

To assess the effects on the peripheral versus resident immune response, a CD45 marker was used. CD11b+/CD45+ cells were selected and separated into discrete populations of high and low CD45 expression; we identified these cell populations as infiltrating macrophages and resident microglia respectively, as previously done by others [41–44], but recognize that other cell types may contribute to these populations (lymphocytes, neutrophils, natural killer cells). Next, a quadrant system was created paring KOR versus CD86 (M1 marker) or KOR versus CD206 (M2 marker) in CD45-high and CD45-low cell populations.

### Magnetic-activated cell sorting (MACS)

For Western blot analysis, microglia and macrophages were separated from a heterogeneous mixture of cells (i.e. inflammatory cells, glia, and neurons) using MACS. The single-cell suspension obtained from spinal tissue was incubated for 15 min with anti-CD11b magnetic beads (Miltenyi Biotec, Germany). After incubation, the suspension was rinsed with 10% BSA to remove unattached CD11b beads. The cells were resuspended in 3 mL of 10% BSA and passed through magnetic separation columns (Miltenyi Biotec, Germany) where CD11b+ cells were trapped while all other cells flowed freely out of the column. Unlabeled cells were collected as they exited the column as the “negative fraction”. The “positive fraction” or CD11b+ cells were extracted from the column by removing the magnetic separation column from the magnetic stand and flushing the cells out using the supplied plunger and 10% BSA.

### Western blots

**Fig. 3:**
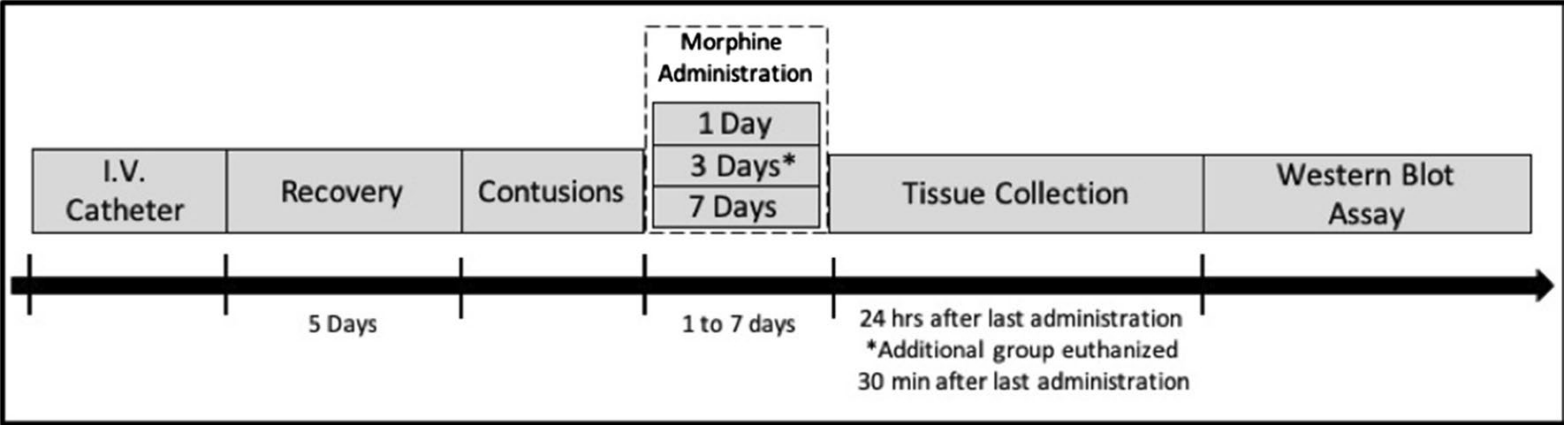
Timeline for Western blot experiments

Western blots were utilized as a semi-quantitative assay of proteins pertaining to opioid receptor signaling pathways of interest (Fig. 3). Tissue for Western blot analysis was collected either 30-min after the last drug administration to look at effects while morphine was pharmacologically active, or 24-h after the last drug administration to examine long-term effects of morphine. The isolated CD11b+ (microglia, macrophages) effluent fractions obtained after MACS separation was collected. Cells were incubated in 1X RIPA (Millipore Sigma, Burlington, MA, USA) with 1% Halt Protease Inhibitor Cocktail buffer (Thermo Fisher Scientific, Waltham, MA, USA) at 4 °C for 30 min. Cells were centrifuged (20,000×*g* for 10 min at 4 °C) and the supernatant containing the protein was extracted for Western blot analyses. The protein concentration was calculated using the Pierce BCA Protein Assay Kit and measured with a NanoDrop spectrophotometer. Samples from 2 rats belonging to the same treatment group were pooled together to obtain sufficient protein to perform the analysis. In this study, we pooled subjects to obtain sufficient protein from CD11b+ cells extracted from the control subjects (sham-saline rats) to perform Western blot analysis. To maintain consistency in sample preparation across all treatment groups, we pooled subjects across all groups. While we cannot describe differences between single subject results and pooling of samples, as we did not conduct those experiments, any variability introduced by pooling of samples would be consistent across groups, allowing for balanced between-group comparisons. 15 ug of protein (mixed with 5 uL of loading dye, 1 ug of reducing agent, and Milli-Q water) were loaded onto 4–12% polyacrylamide gel, separated by SDS-PAGE (at constant voltage of 100 V for 200 min), and transferred to a PVDF membrane with the iBlot™ Dry Blotting System (Invitrogen by Thermo Fisher Scientific, Waltham, MA, USA). The membranes were rinsed with 1X TBST and left to dry overnight. The PVDF membrane was reactivated by adding 2 mL of 100% methanol for 1 min and rinsing it with Milli-Q-grade water. Membranes were stained with REVERT Total Protein Stain (LI-COR Biosciences, Lincoln, NE, USA) for normalizing target signals (Fig. 10A). Staining of target proteins was done using the iBind™ Flex Western System (Invitrogen by Thermo Fisher Scientific, Waltham, MA, USA) and antibodies according to Table 2. All fluorescent detection of Western blots was done with a LI-COR detection system and data was analyzed using Empiria Studio™ Software (LI-COR Biosciences, Lincoln, NE, USA).

**Table 2:** List of antibodies used for Western blots

Antibody	Concentration	Manufacturer	Catalog No
*Unconjugated primary antibodies*			
B-arrestin	1:100	ThermoFisher, Waltham, MA, USA	39-5000
p38	1:500	Abcam, Cambridge, UK	ab31828
Dynorphin	1:100	Abcam, Cambridge, UK	ab82509
ERK 1/2	1:500	Cell Signaling, Danvers, MA, USA	9102S
Kappa Opioid Receptor	1:50	ThermoFisher, Waltham, MA, USA	44-302G
	1:1000		
*Secondary antibodies*			
IRDye 680RD Goat Anti Mouse	1:2000	LI-COR Biosciences, Lincoln, NE	926-32210
IRDye 800CW Goat Anti Rabbit	1:2000	LI-COR Biosciences, Lincoln, NE	827-08365

### Statistical analysis

Locomotor scores were analyzed using a two-way repeated measures ANOVA to examine the effect of drug treatment. Only contused rats treated for the full 7 days were used in these analyses. Three-way ANOVAs were used to compare pain reactivity thresholds, with drug treatment and surgery as between subjects variables and day of testing as the repeated factor. Again, only rats treated for 7 days were included in these analyses. When main effects were found to be significant, a Holm-Sidak test was used for post-hoc analyses. Two-way ANOVAs were used to compare the effects of surgery and drug treatment on cell expression and protein expression of CD11b+ cells.

## Results

### Effects of morphine on locomotor and sensory recovery

#### Effects of intravenous morphine on locomotor recovery

**Fig. 4:**
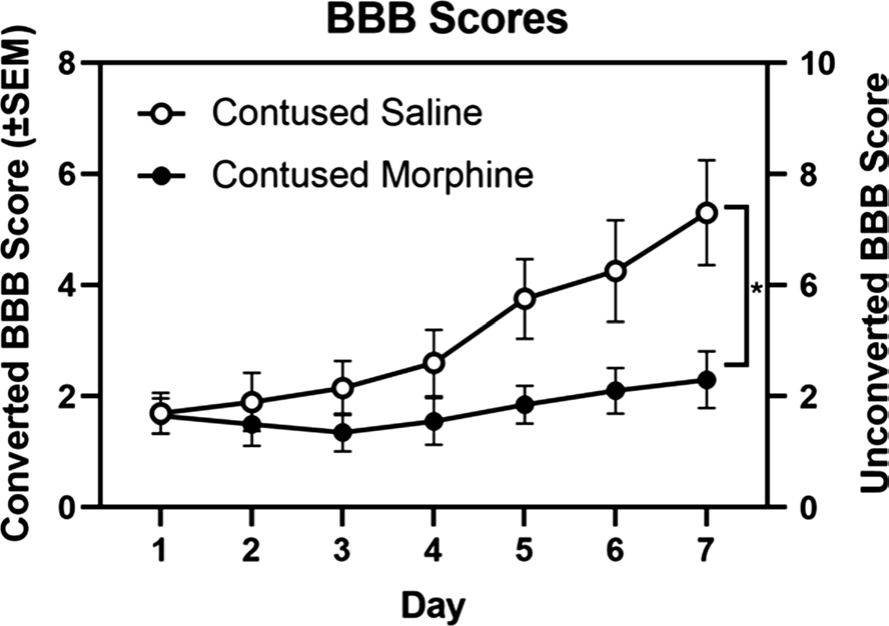
Effects of intravenous morphine on locomotor recovery. BBB scores were balanced across groups on day 1 post-injury. Morphine significantly undermined locomotor recovery in contused rats after 7 days of IV administration. Results shown as Mean ± S.E.M. *p < 0.05, n = 10

To assess the effects of morphine on recovery, locomotor behavior was scored daily using the BBB scale [35]. Figure 4 shows mean converted BBB scores (± S.E.M) for the first seven days after surgery. Locomotor scores on day one prior to drug treatment did not differ significantly between contused saline (1.70 ± 0.35) and contused morphine (1.65 ± 0.30) rats.

A two-way repeated measures ANOVA showed a main effect of day post-surgery (*F* (6, 108) = 19.72, *p* < 0.001), but not of treatment (*F* (1, 18) = 3.622, *p* = 0.07), on BBB scores. However, replicating our previous studies, our analysis revealed a significant interaction between day post-surgery and treatment (*F* (6, 108) = 7.694, *p* < 0.001). Saline-treated rats had significantly higher BBB scores than morphine-treated rats on days 6 and 7 post-surgery (*t* (126) = 2.739, *p* < 0.05; *t* (126) = 3.822, *p* < 0.05, respectively). Vehicle control rats recovered to an average converted BBB score of 5.30 ± 0.89, while morphine-treated rats recovered to an average converted BBB score of 2.30 ± 0.49. All sham rats, irrespective of treatment, had a converted BBB score of 12 (unconverted BBB score of 21) throughout the 7 day post-surgery assessment period.

#### Effects of morphine on sensory and mechanical reactivity

**Fig. 5:**
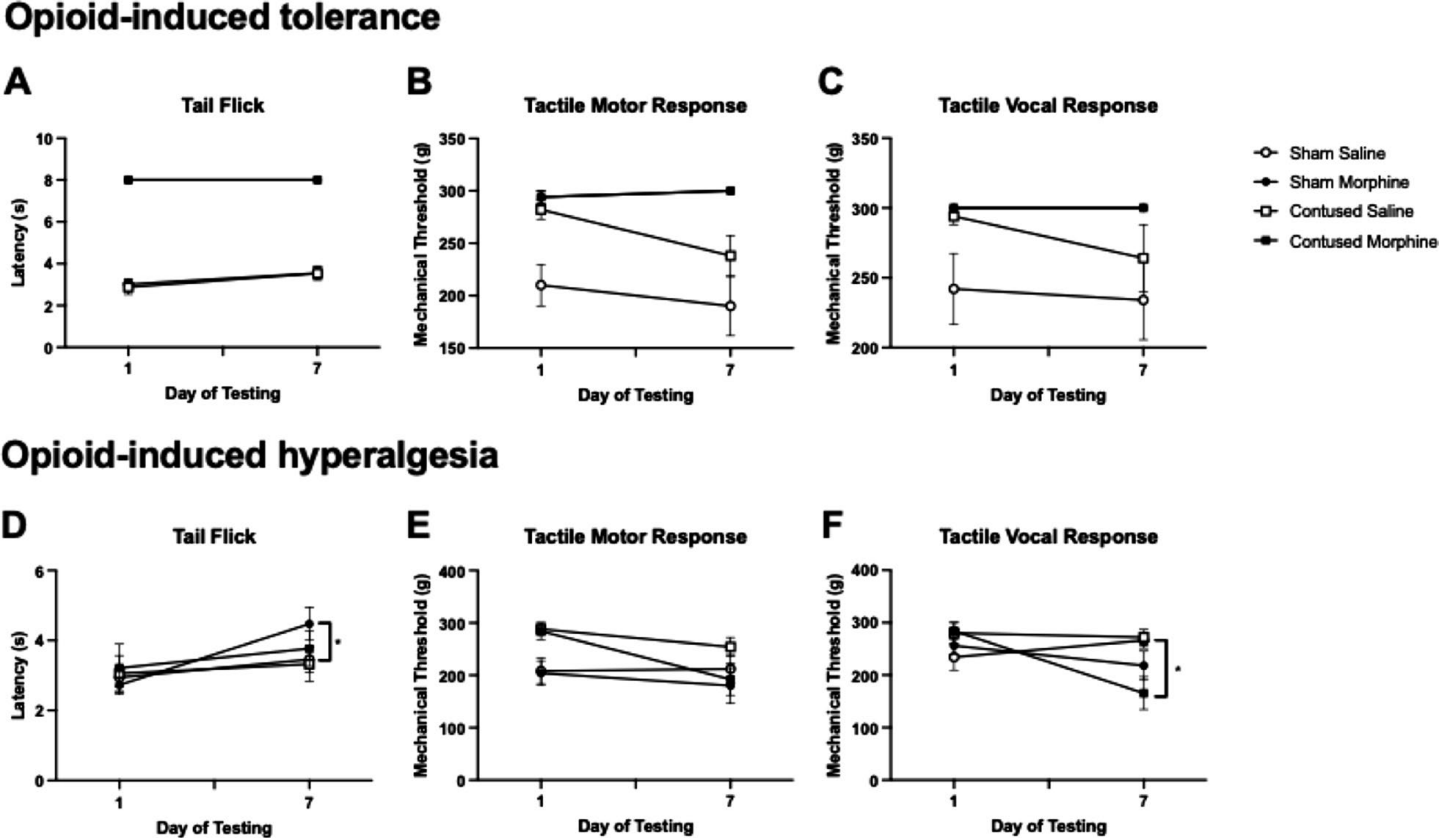
Effects of morphine on thermal and tactile recovery. Morphine produced robust analgesia on thermal (**A**) and tactile (**B**, **C**) reactivity. There was no reduction in the efficacy of morphine across days on either sensory test. Conversely, the development of hyperalgesia was observed by day 7 on the tactile reactivity (**E**, **F**) test but not for thermal reactivity (**D**). Contused rats treated with morphine displayed significantly lower thresholds for vocal (**F**), and a trend for reduction of motor (**E**), responses on the tactile reactivity test. Results shown as Mean ± S.E.M. *p < 0.05, n = 10

Figure 5 illustrates the effects of morphine on sensory recovery for the first seven days following sham or contusion injury. Sensory recovery was evaluated by assessing thermal (tail-flick test) and mechanical reactivity (von Frey filaments) on the first and last day of treatment, immediately before and 30-min after the intravenous administration of morphine.

##### Assessment of the analgesic efficacy of morphine

Morphine produced robust analgesia on the thermal reactivity test that persisted across the 7 days of administration (Fig. 5A). Both sham and contused animals treated with morphine reached the maximum 8-s tail-flick latency. A 3-way ANOVA revealed a significant main effect of drug treatment (*F* (1, 36) = 1092, *p* < 0.001). There were no effects of surgery (*F* (1, 36) = 0.05, *p* = 0.81) or day post injury (*F* (1, 36) = 2.97, *p* = 0.09), and no significant Surgery × Drug treatment interactions.

Similarly, morphine produced robust analgesia on the von Frey test of mechanical reactivity at all timepoints. A 3-way ANOVA revealed a main effect of surgery (*F* (1, 36) = 8.95, *p* < 0.05), a main effect of drug treatment (*F* (1, 36) = 44.62, *p* < 0.001), and a Surgery × Drug treatment interaction (*F* (1, 36) = 8.95, *p* < 0.05) on the motor response to mechanical stimulation (Fig. 5B). Sham rats showed higher sensitivity to tactile stimulation than contused rats across all days. Morphine administration also resulted in less vocal responses to mechanical stimulation across all testing days (Fig. 5C). There was a main effect of drug treatment (*F* (1, 36) = 10.79, *p* < 0.001), and no effect of surgery or significant interaction between drug treatment and surgery. We did not see evidence of tolerance to morphine developing across days. Analgesia on the thermal reactivity and mechanical stimulation tests persisted across the seven days of administration (Fig. 5A–C).

##### Assessment of opioid-induced hyperalgesia

Thermal and mechanical reactivity were also assessed on Days 1 and 7 post injury, prior to drug administration, to examine the development of opioid-induced hyperalgesia. There were no significant differences in tail-flick reactivity between conditions on Day 1 post-injury. Across the 7 days post-injury, however, there was a main effect of day of testing (*F* (1, 36) = 7.21, *p* < 0.05) but no effect of surgery, drug treatment, or significant interactions (Fig. 5D). Post hoc comparisons, revealed that the latency to tail-flick was increased in morphine-treated sham rats from Day 1 to Day 7 after injury (*t* (36) = 3.05, *p* < 0.05). Tail-flick latency did not change for the rest of the treatment groups across days.

Conversely, evidence of opioid-induced hyperalgesia was observed on the mechanical reactivity test (Fig. 5, E–F). As with the assessment of tolerance, contused animals showed less motor reactivity to mechanical stimulation compared to sham rats (*F* (1, 36) = 11.43, *p* < 0.001). Additionally, statistical analyses of the vocal responses to mechanical stimulation revealed a main effect of day of testing (*F* (1, 36) = 6.810, *p* < 0.05), where Day 7 vocalization scores were significantly lower relative to Day 1 scores. There were also significant interactions between day of testing and surgery (*F* (1, 36) = 3.452, *p* < 0.05), and between day of testing and drug treatment (*F* (1, 36) = 12.64, *p* < 0.001). Post hoc analyses show that these effects were mainly driven by changes in the threshold for vocalizations in contused animals treated with morphine. As shown in Fig. 5F, vocalization reactivity thresholds were significantly decreased from Day 1 to day 7 (*t* (36) = 4.69, *p* < 0.001) in the morphine-treated SCI rats, indicating the development of opioid-induced hyperalgesia.

### Morphine increases the total number of microglia and macrophages

Based on our previous studies [15], we hypothesized that morphine enhances inflammation and exacerbates the secondary injury cascade by increasing the number of immune cells at the injury site. To test this, we used flow cytometry to compare the expression of microglia and macrophages at the SCI lesion site after repeated i.v. administration of morphine versus vehicle. First, we quantified the total number of CD11b+ cells as an indirect measure of inflammation. To be considered positive (+), cell populations were selected using a gate that contained < 1% CD11b+ unstained cells.

**Fig. 6:**
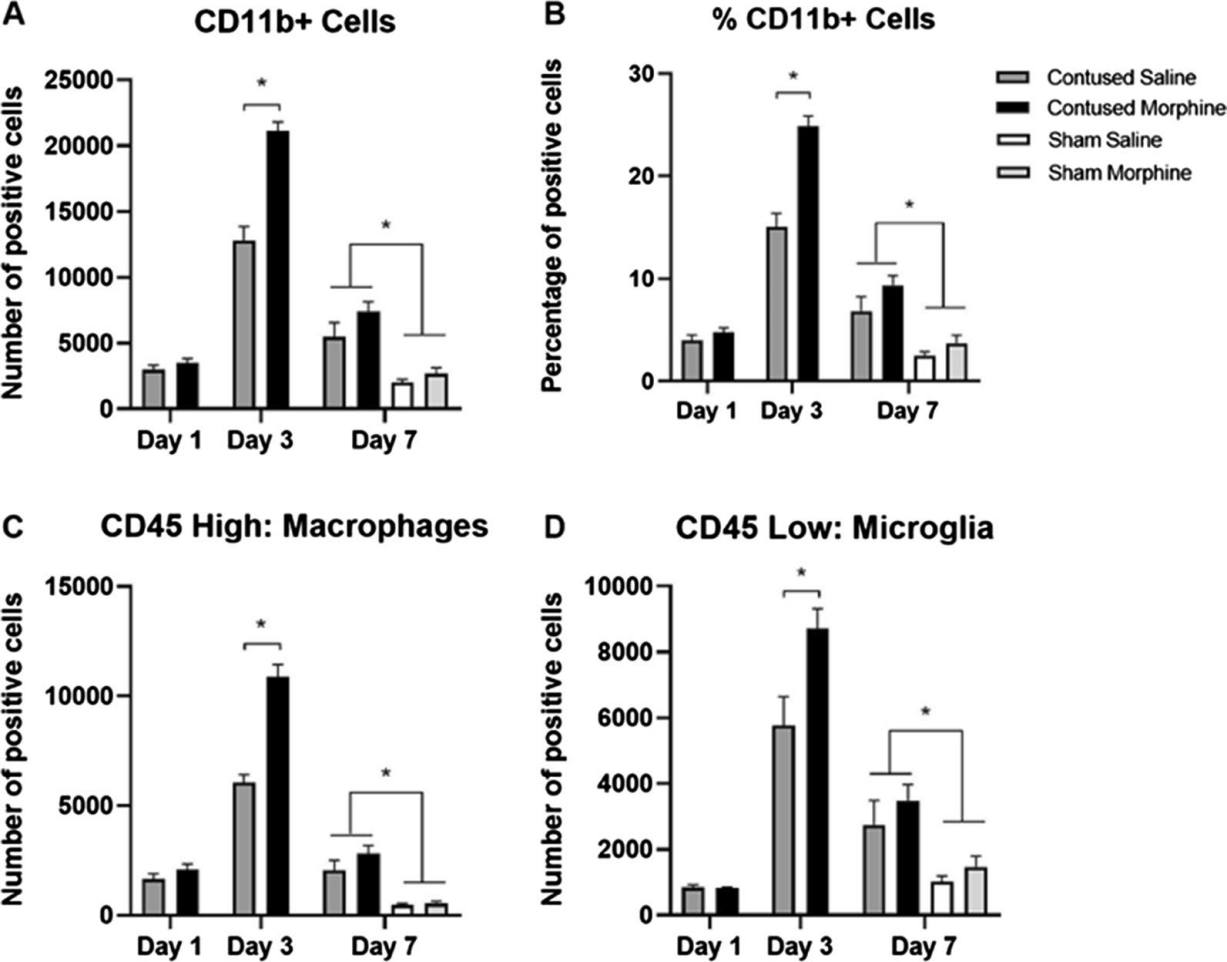
Quantification of microglia and macrophages in CD86 set using flow cytometry. The contusion injury significantly increases the number of CD11b total positive cells (**A**), percentage of CD11b positive cells (**B**), total number of macrophages (**C**), and total number of microglia (**D**) at the site of injury relative to a sham surgery. After 3 days of morphine administration, contused animals also had a significantly higher total number of CD11b positive cells (**A**), percentage of CD11b positive cells (**B**), total number of macrophages (**C**), and total number of microglia (**D**) compared with vehicle SCI controls. There was no significant effect of treatment with 1 or 7 days of morphine administration. Results shown as Mean ± S.E.M. *p < 0.05, n = 5–6

Focusing on the M1 antibody panel, the contusion injury significantly increased the number and percentage of CD11b+ cells at 7 days post injury, compared to sham surgery (*F* (1, 20) = 33.54, *p* < 0.001 and *F* (1, 20) = 27.82, *p* < 0.001, respectively), regardless of drug treatment. Importantly, an effect of morphine on the number of CD11b+ cells was also observed after just 3 days of administration. At this timepoint there was a significant increase in the number of CD11b+ cells (*t* (8) = 6.67, *p* < 0.001, Fig. 6A and B) in morphine-treated SCI rats compared to their saline counterparts. The same effects of surgery and day post injury (*F* (1, 20) = 7.080, *p* < 0.05 and *F* (1, 20) = 7.392, *p* < 0.05) were seen in analyses of the M2 antibody panel (see Additional file 2). Again, there was significant increase in the number of CD11b + cells (*t* (8) = 4.768, *p* < 0.001), macrophages (*t* (8) = 4.691, *p* < 0.001), and microglia (*t* (8) = 3.492, *p* < 0.001) on Day 3 post injury in the morphine-treated rats.

To assess the effects on the peripheral versus resident immune response, a CD45 marker was used. CD11b+/CD45+ cells were selected and separated into discrete populations of high and low CD45 expression; we identified these cell populations as infiltrating macrophages (CD11b+/CD45^High^) and resident microglia (CD11b+/CD45^Low^) respectively, as previously done by others [41–44], but recognize that other cells may have been included in these populations. Similar to total CD11b+ quantification, after SCI and 3 days of morphine administration our analysis of the M1 antibody panel shows a significant increase in the number of both macrophages (*t* (8) = 7.03, *p* < 0.001), and microglia (*t* (8) = 2.79, *p* < 0.05, Fig. 6C and D, respectively), compared to vehicle-treated rats. Replicating this finding, for the M2 panel, morphine-treated SCI rats had an increased number of macrophages (*t* (8) = 4.691, *p* < 0.001) and microglia (*t* (8) = 3.492, *p* < 0.001) at the injury site, compared to vehicle-treated rats. There was no effect of treatment with 1 or 7 days of morphine on the number or percentage of CD11b+ macrophages (CD11b+/CD45^High^), or microglia (CD11b+/CD45^Low^). Together, these results show that morphine administration produces a transient, but dramatic surge in the number of immune cells, including macrophages and microglia, in a rodent model of SCI.

#### Effect of morphine on CD86^+^ microglia/macrophages

**Fig. 7:**
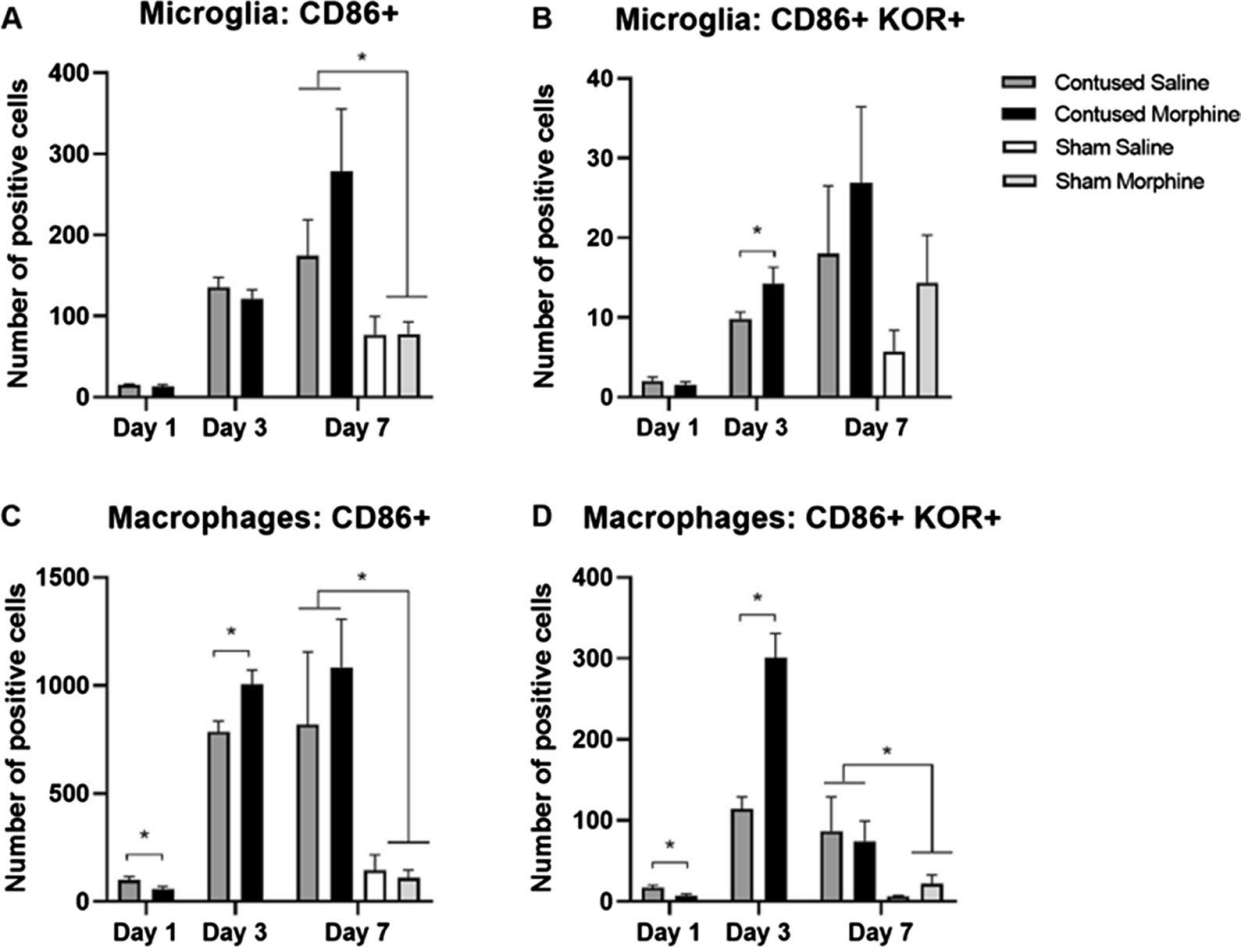
Quantification of microglia/macrophages expressing CD86 (M1 marker) and KOR. Morphine significantly increases the number of CD86+ macrophages (**C**) in contused rats after 3 days of administration but had no effect on CD86+ microglia at any timepoint (**A**). However, 3 days of morphine administration significantly increased the number of CD86 + microglia and macrophages expressing KORs (**B** and **D**). Results shown as Mean ± S.E.M. *p < 0.05, n = 5–6

Immune cells like microglia and macrophages are highly heterogenous in phenotype and function. Thus, after quantifying the overall expression of cells, we further examined the phenotype of the cells present at the site of injury, quantifying the number of cells within each population expressing the CD86 marker (M1 panel). Our statistical analyses show there were no significant changes in the overall number of CD86+ microglia (*t* (8) = 0.85, *p* = 0.4226, Fig. 7A) at any timepoint. However, morphine had divergent effects on CD86+ macrophages at different timepoints. Contused rats treated with morphine had significantly less CD86 + (Fig. 7C) macrophages after 1 day of administration (*t* (10) = 2.33, *p* < 0.05). However, after 3 days of morphine administration the overall number of CD86+ macrophages significantly increased (*t* (8) = 2.658, *p* < 0.05, Fig. 7C) relative to vehicle-treated contused rats. Not surprisingly, there was a main effect of surgery on CD86 expressing macrophages, with contused rats exhibiting significantly higher numbers of CD86+ macrophages than shams on day 7 post injury (*F* (1, 20) = 16.07, *p* < 0.001, Fig. 7C).

Additionally, we previously found that activation of KORs is both sufficient and necessary to mediate the negative effects of morphine on motor recovery in the rat model of SCI [12, 13]. To explore whether morphine administration altered the expression of opioid receptors on specific subpopulations of immune cells we quantified KOR expression within the CD86 + subpopulations of microglia and macrophages. Our results show that morphine significantly increased the number of CD86 + microglia expressing KORs after 3 days of drug administration (*t* (8) = 1.92, *p* < 0.05) in comparison to saline-treated animals (Fig. 7B). There was an additional main effect of surgery on the expression of KORs after 7 days of treatment, with contused rats exhibiting significantly higher numbers of KOR + microglia (*F* (1, 20) = 5.26, *p* < 0.05) compared to sham rats, irrespective of treatment (Fig. 7B). There were no other significant effects or interactions on Days 1 and 7. For macrophages, after 1 day of morphine administration, there is a significant decrease in the overall number (Fig. 7D) of CD86 + cells expressing KORs (t (10) = 2.574, p < 0.05). Again, this effect is reversed after 3 days of administration when there is a significant increase in the overall number of CD86 + macrophages expressing KORs relative to vehicle treated controls (t (8) = 5.645, p < 0.0001). There is also a main effect of surgery on the expression of KOR on CD86+ macrophages observed after day 7, with contused rats exhibiting a higher number of CD86+ macrophages expressing KORs (F (1, 20) = 6.96, p < 0.05) compared to sham rats (Fig. 7D). These data show that both SCI and morphine administration increase the expression of CD86+ macrophages around the center of the lesion, as well as increasing the number of CD86+ macrophages expressing KORs.

#### Effect of morphine on CD206+ microglia/macrophages

**Fig. 8:**
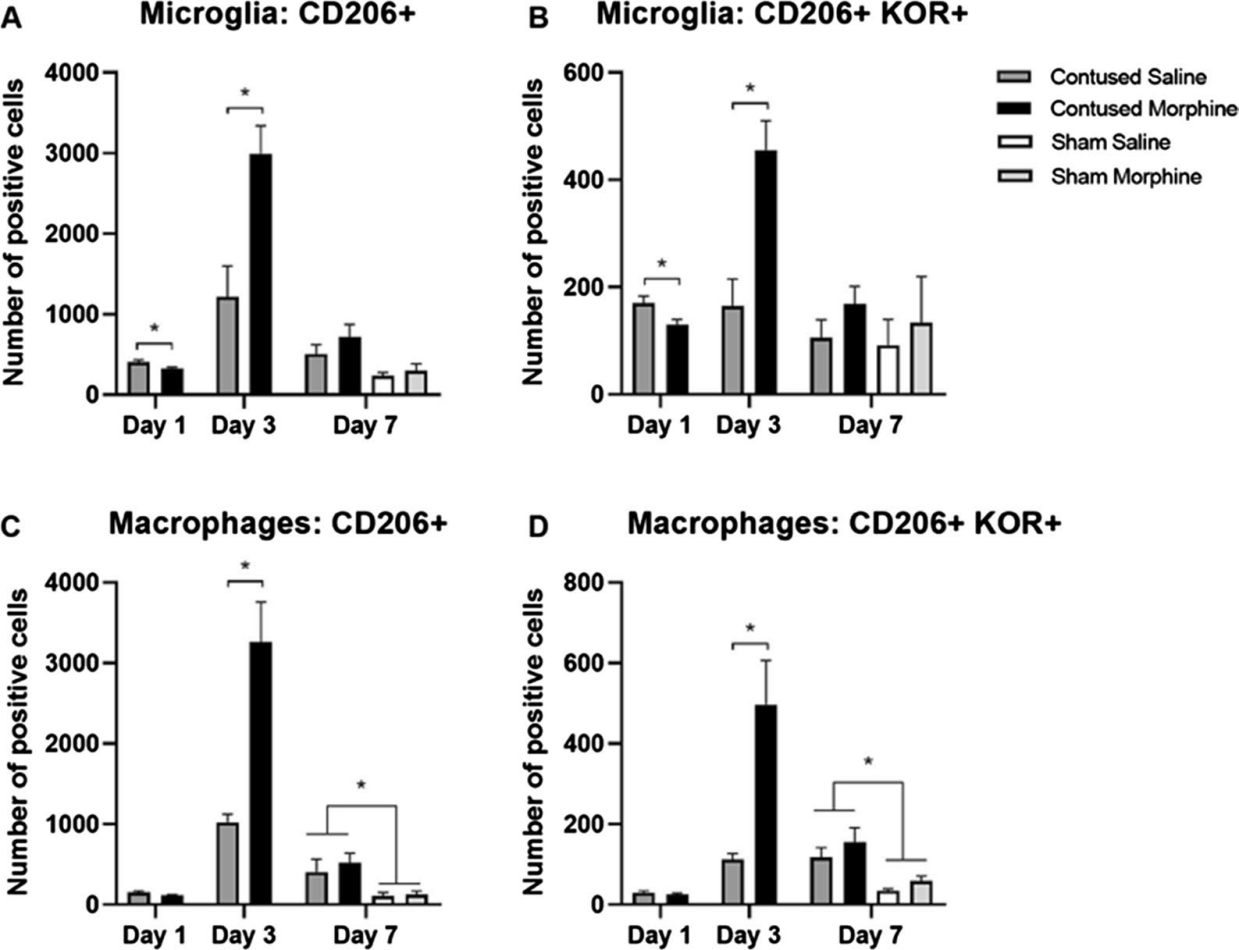
Quantification of microglia/macrophages expressing CD206 (M2 marker) and KOR. Morphine significantly increases the number of CD206+ microglia (**A**) and CD206+ macrophages (**C**) after 3 days of administration. Morphine administration also increases the number of CD206 + microglia and macrophages expressing KORs (**B** and **D**). Results shown as Mean ± S.E.M. *p < 0.05, n = 6

Another subpopulation of glia cells that are relevant within the context of SCI are CD206 expressing cells. Transplantation of CD206+ microglia and macrophages into the site of lesion after an SCI has been reported to promote recovery of function in rodents [45–47]. Thus, as we did before, we used flow cytometry to quantify the number of glia expressing the CD206 and KOR markers (M2 panel, Fig. 8). After 1 day of administration, saline-treated rats had a modest but significantly higher number of CD206 + microglia (*t* (8) = 2.698, *p* < 0.05, Fig. 8A) compared to morphine-treated rats. Conversely, after 3 days of morphine administration there was a significant increase in the overall number of CD206+ microglia (*t* (8) = 3.456, *p* < 0.001, Fig. 8A) and CD206+ macrophages (*t* (8) = 4.423, *p* < 0.001, Fig. 8C) in morphine-treated rats versus saline controls.

Similarly, after 1 day of treatment, saline-treated rats showed significantly higher numbers of CD206+ microglia expressing KORs (*t* (8) = 2.659, *p* < 0.05, Fig. 8B) compared to morphine-treated rats. Again, this effect reversed after 3 days of morphine, compared to vehicle, administration when there was an increase in the total number of CD206+ microglia expressing KORs (*t* (8) = 3.905, *p* < 0.001), and an increase in the overall number of CD206+/KOR+ macrophages (*t* (8) = 4.926, *p* < 0.001, Fig. 8D). There was an additional main effect of surgery, where contused rats showed significantly higher numbers of CD206+ macrophages (F (1, 20) = 11.04, p < 0.001, Fig. 8B), and CD206+ KOR+ macrophages (F (1, 20) = 15.70, p < 0.001, Fig. 8D) compared to sham animals. There were no other significant main effects or interactions for days 1 and 7. Therefore, similar to the CD86+ population, our results show that SCI alone increases the number of CD206+ macrophages/microglia. Morphine administration further increases the expression of CD206+ cells after SCI, as well as increasing the number of CD206+/KOR+ macrophages and microglia. Interestingly, the morphine effects seem to be larger in CD206+ cells compared to CD86+ cell populations.

### Effect of morphine on protein expression in CD11b+ cells

In addition to increasing the number of microglia and macrophages, we hypothesized that morphine administration may change the function of these cells after SCI. To address this, we used Western blot analyses to examine whether morphine-induced activation of microglia and macrophages after SCI engages signaling pathways associated with the production of pro-inflammatory cytokines and neurotoxicity.

#### Effect of morphine on protein expression 30-min post-administration

**Fig. 9:**
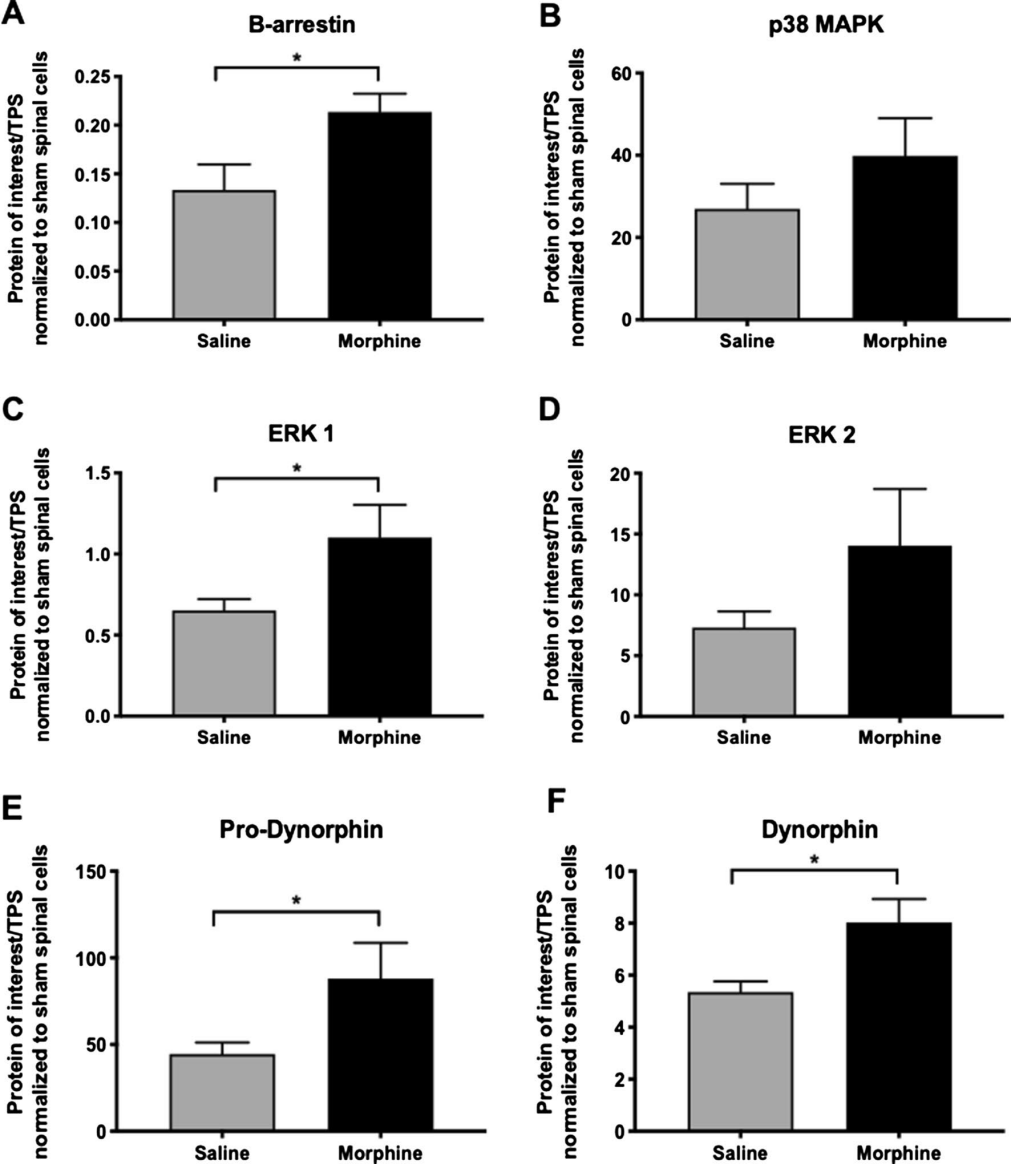
Quantification of β-arrestin pathway protein, Dynorphin, and Pro-Dynorphin in CD11b+ cells from center of lesion 30-min after 3 days of drug administration. Morphine administration significantly increases the expression of β-arrestin (**A**) and ERK 1 (**C**) in contused rats compared to their saline-treated counterparts. Morphine administration significantly increases the expression of pro-dynorphin (**E**) and dynorphin (**F**) in contused rats compared to their saline-treated counterparts. There was a trend towards higher expression of p38 MAPK (**B**) and ERK 2 (**D**) with morphine treatment but no significant effects were found. Loading control: protein extracted from CD11b+ cells from spinal cord tissue from sham rats treated with saline. Results shown as Mean ± S.E.M. *p < 0.05, n = 6

Activation of classic opioid receptors (ORs) on immune cells has been shown to induce the inflammatory β-arrestin pathway [18, 22, 23]. Thus, we used Western blot analysis to estimate changes in the concentration of proteins downstream of β-arrestin in CD11b+ cells (microglia and macrophages), 30 min post-morphine administration. Quantification revealed a significant increase in β-arrestin expression in morphine-treated rats (*t* (10) = 2.49, *p* < 0.05) and ERK 1 (*t* (10) = 2.10, *p* < 0.05) relative to saline-treated contused controls (Fig. 9A and C). There was also a trend toward an increase in p38 MAPK and ERK 2 expression in morphine-treated rats (Fig. 9B and D), but these effects were not significant (t (10) = 1.17, p < 0.13 and t (10) = 1.38, p < 0.09, respectively). OR activation also induces ERK 1/2 which is a kinase responsible for CREB upregulation of dynorphin gene expression [48]. Supra-physiological levels of dynorphin within the spinal cord can induce neurotoxicity and cause motor dysfunction [49], so we used Western blot analysis to look at components of this pathway. Morphine administration significantly increased the expression of pro-dynorphin (t (10) = 2.014, p < 0.05) and dynorphin (t (10) = 2.715, p < 0.05), compared to saline-treated rats (Fig. 9E and F).

#### Effect of morphine on protein expression 24-h post-administration

**Fig. 10:**
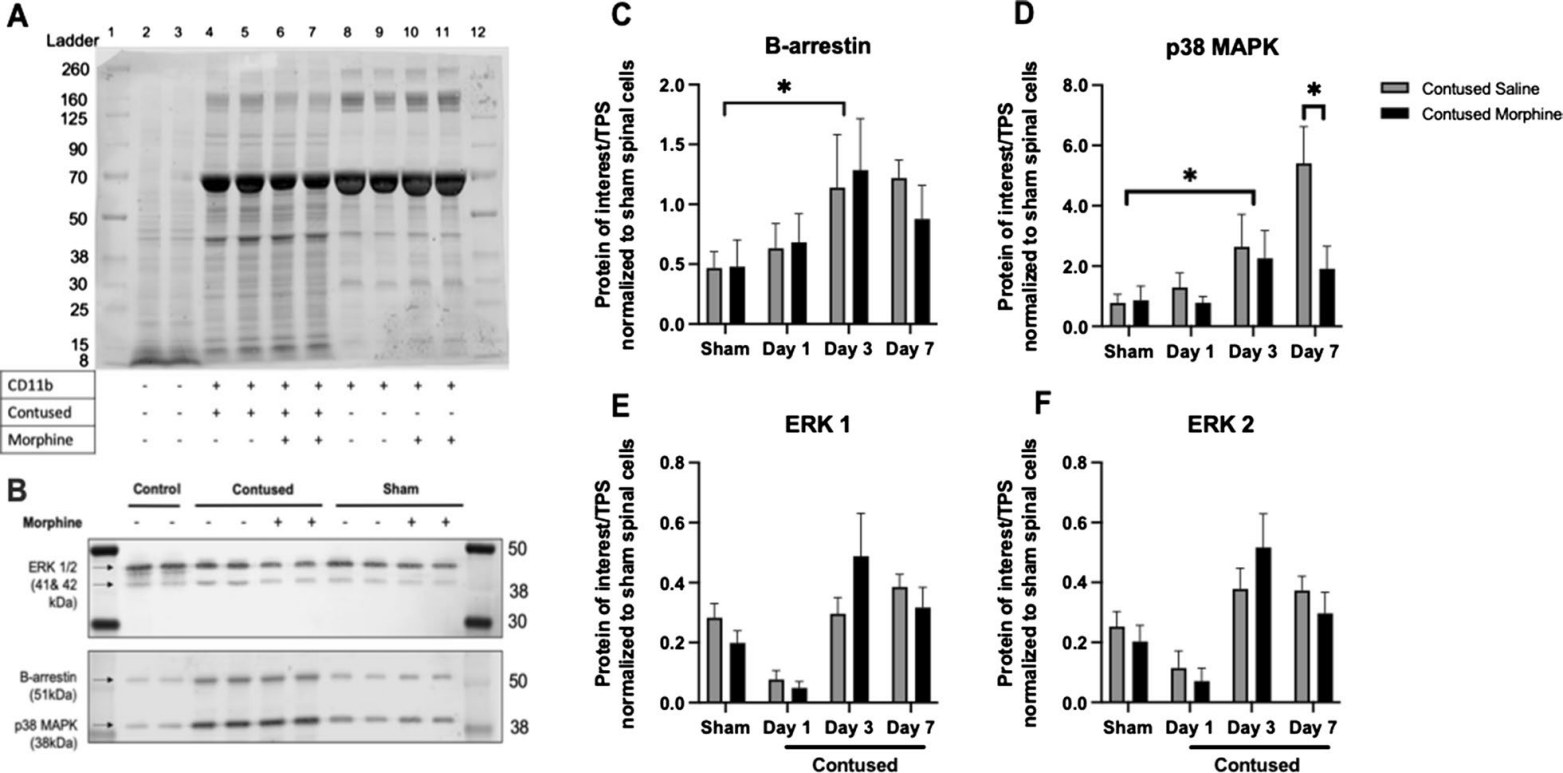
Quantification of β-arrestin pathway proteins in CD11b+ cells from center of lesion 24-h after morphine administration. Total Protein Stain as internal loading control for normalization of target signals (**A**). Protein collected from rats treated for 3 days with i.v. drug administration. Loading control: protein extracted from whole spinal cord tissue from sham rats treated with saline (lanes 2–3). Western blot image (**B**) protein was collected from rats that received 3 days of consecutive i.v. drug treatment and samples taken 24-h after the last drug dose and processed with LI-COR system. **B** Western blot images were cropped to remove irrelevant sections of the image and display only the proteins of interest. The contusion injury significantly increased the expression of β-arrestin (**C**) and p38 MAPK (**D**) on day 3. Additionally, morphine significantly decreases the expression of p38 MAPK by day 7 compared to saline-treated rats (**D**).  There was a trend towards higher expression of ERK 1 (**E**) and ERK 2 (**F**) with on day 3 of morphine treatment but no significant effects were found. Results shown as Mean ± S.E.M. *p < 0.05, n = 6

Twenty four hours post morphine administration (3 days of administration), there was also a main effect of surgery with SCI significantly increasing the expression of the β-arrestin protein compared to sham surgery (*F* (1, 20) = 4.85, *p* < 0.05), irrespective of drug treatment (Fig. 10B and C). Similarly, the contusion injury significantly increased the expression of p38 MAPK in CD11b + cells compared to the sham surgery (*F* (1, 20) = 4.57, *p* < 0.05) irrespective of treatment (Fig. 10D). Surprisingly, after 7 days of drug administration there was a significant decrease in the expression of p38 MAPK in morphine-treated rats (*t* (10) = 2.465, *p* < 0.05) as compared to saline-treated animals (see Additional file 4).

**Fig. 11:**
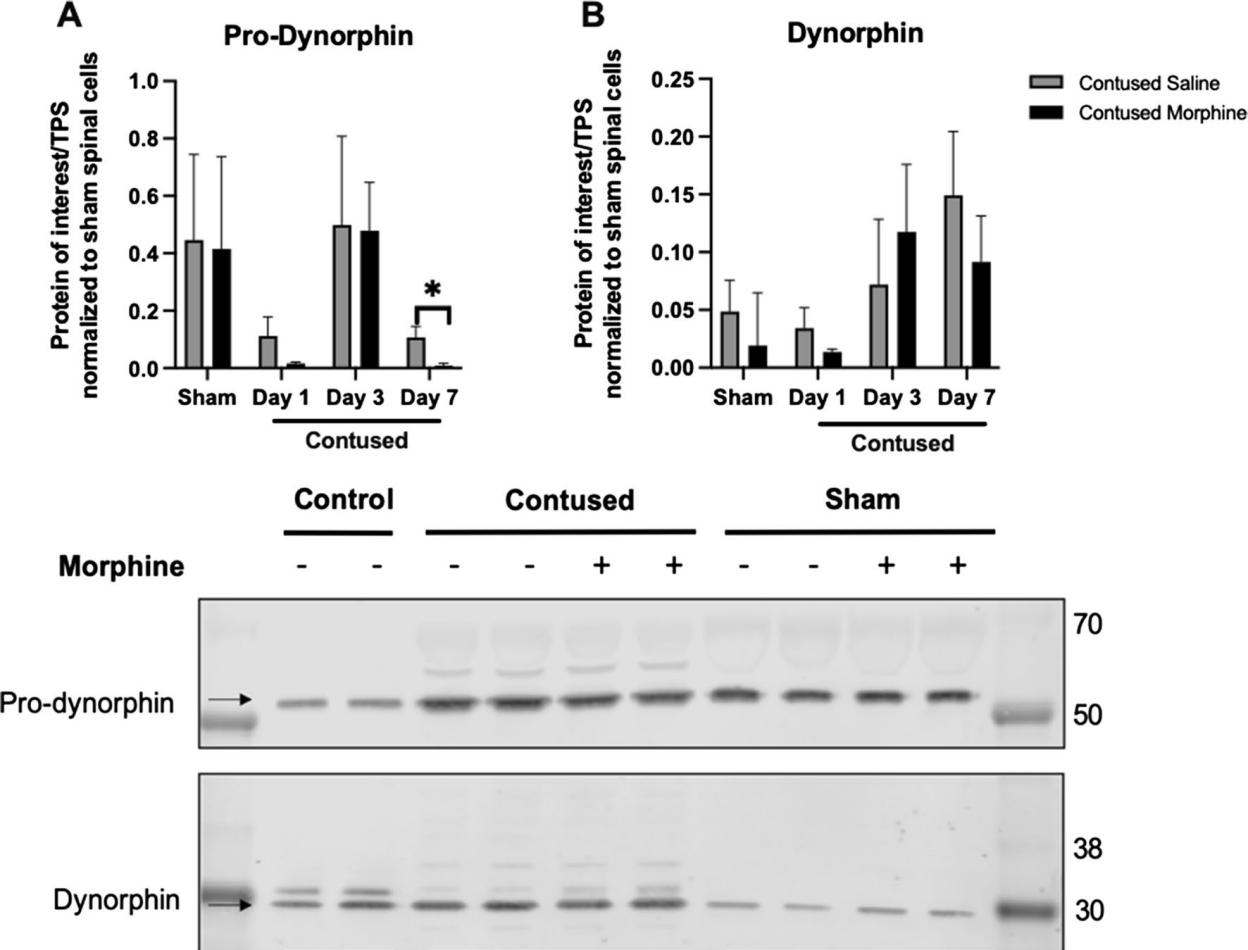
Quantification of Dynorphin and its precursor Pro-Dynorphin in CD11b+ cells taken from center of lesion 24-h after administration. Morphine administration significantly decreases the expression of pro-dynorphin compared to saline-treated subjects by day 7 (**A**) but not the expression of dynorphin (**B**). Western blot image (**C**) protein was collected from subjects that received 3 days of consecutive i.v. drug treatment. Samples taken 24-h after the last drug dose and processed with LI-COR system. (**C)** Western blot images were cropped to remove irrelevant sections of the image and display only the proteins of interest. Loading control: protein extracted from whole spinal cord tissue from sham subjects treated with saline (lanes 2–3). REVERT Total protein stain used as internal control. Tissue used for Western blot analysis was collected 24-h after the last drug-administration. Results shown as Mean ± S.E.M. *p < 0.05, n = 6

While there was a trend toward an increase in protein expression of ERK 1 and ERK 2 in the CD11b + cells extracted from the rats treated with morphine for 3 days (*t* (10) = 1.32, *p* = 0.10 and *t* (10) = 1.06, *p* = 0.16, respectively), there were no main effects of surgery or drug-treatment at any time point (Fig. 10E and F). There were also no significant effects of surgery on the expression of pro-dynorphin or dynorphin (See Additional files 3 and 4). However, similar to p38 MAPK levels, there was a significant decrease in the expression of pro-dynorphin after day 7 in morphine-treated rats (*t* (10) = 2.502, *p* < 0.05) compared to saline-treated rats (Fig. 11A and C). There were no additional effects of treatment for Days 1 or 3 of treatment (see Additional file 3). Our statistical analyses did not show main effects of treatment for dynorphin on any of the test days (Fig. 11B).

## Discussion

Intravenous morphine administration significantly altered the immune response after SCI. We found that morphine increased the total number of activated microglia and macrophages at the site of the spinal cord injury. It also increased the expression of both CD86+ and CD206+ cells. Further, our data shows that morphine changes the function of microglia and macrophages, by engaging the β-arrestin signaling pathway which is linked to cell death and inflammation. These effects of morphine may underlie the deficits in recovery that we see in the in vivo model. Indeed, replicating previous studies, we showed that intravenous morphine undermines locomotor recovery and increases hyperalgesia.

After SCI, CD86 has been often used as a marker to identify cells that generally have neurotoxic or pro-inflammatory functions. CD206+ macrophages, on the other hand, are considered anti-inflammatory, and treatments that promote their expression after injury are reported to improve locomotion, increase neuronal survival, and result in greater re-myelination [46, 50–53]. Given that morphine increases the expression of pro-inflammatory cytokines around the lesion [9, 15, 54], we had originally hypothesized that morphine would also increase the expression of CD86+ cells and decrease the number of CD206+ macrophages. However, the results from this study suggest that the complex mechanism through which morphine increases inflammation might include an increase in the expression of more than one phenotype of macrophages. While we found that morphine did, in fact, increase the total number of CD86+ macrophages present at the lesion, we observed the most robust increase in the total number of CD206+ microglia and macrophages with morphine administration.

In this study different CD11b antibodies were used to optimize our first (CD86) and second (CD206) phenotype panels based on the commercial availability of the antibodies of interest and their conjugates. As such, the gating strategy was lightly adjusted to accommodate for a higher autofluorescence baseline on the first panel precluding a direct comparison between the number of positively stained cells between both sets. However, when we compare cell expression to our control groups within the same staining set, our data shows that morphine administration after SCI increased CD86+ macrophages by only a modest amount compared to saline, and that there were no changes in CD86+ microglia expression with drug treatment. On the other hand, we found a three-fold increase in CD206+ microglia and macrophages in morphine-treated contused rats compared to their saline counterparts. Others have also reported that short-term administration of morphine (3 days) reduces the number of pro-inflammatory macrophages and increases anti-inflammatory macrophages in a model of incision-induced inflammation in mice [55]. The authors in this study saw a morphine-induced shift towards CD206+ anti-inflammatory macrophages and reported that blocking this shift reversed morphine-induced analgesia. Other groups have found that the analgesic effects of morphine in a model of neuropathic pain were dependent on an increased release of opioid peptides by CD206+ macrophages [55]. Yet, the consequences of a morphine-induced early surge of CD206+ macrophages on motor recovery after an SCI are not well understood.

Elevated expression of the CD206 marker in macrophages so early after the injury is in direct contrast with the classic timeline reported after SCI. Classic SCI literature reports that pro-inflammatory cells predominate the site of injury immediately following the lesion and outweigh anti-inflammatory cells for up to 2 weeks post-injury, after this period the balance slowly shifts toward the anti-inflammatory or repair phenotype [56, 57]. Yet, in morphine-treated rats we see a dramatic increase in CD206+ microglia and macrophages which are typically described as having tissue-repair associated functions [57]. In the literature, treatments that improve functional recovery boost the number of reparatory cells at the site of injury but they do not suppress the initial inflammatory response. Instead, in these experiments, the authors change the microenvironment around the injury to accelerate the polarization of M0 microglia/macrophages towards the anti-inflammatory phenotype [45, 46]. With this approach, there is a gradual shift in the polarization of cells that slowly occurs over the first week post injury. More recently, studies have also identified an alternate activation state for microglia known as disease-associated microglia (DAM). These microglia are found in different disease states including inflammation and SCI, and are thought to play an important role for functional recovery after SCI [58]. Future studies could expand on the results from this study by exploring how morphine might affect the expression and function of DAM after an SCI, and whether DAM contributes to the negative effects on locomotor recovery we see in our model. In the present study, however, we find that with morphine there is a rapid and sudden increase in the number of CD206+ cells at the injury site 3 days post injury. It is possible that increasing the expression of CD206+ cells too soon following the injury might be detrimental for recovery. Supporting this hypothesis, Poulen et al. [59] showed that reducing microglia proliferation 1-week after SCI improves recovery. Moreover, Pannel et al. [60] report that M2-polarized cells, which are also CD206+, contain and release higher levels of opioid peptides including dynorphin. Of interest, our data also showed that morphine administration further increases the expression of dynorphin and pro-dynorphin in CD11b+ cells at the 30-min collection timepoint.

Prior studies have shown that dynorphin expression increases after an SCI and correlates with injury severity [27, 61–63]. After SCI, low dynorphin levels mediate analgesia via activation of KORs on neurons [64]. However, prolonged exposure to, or supraphysiological concentrations of, dynorphin is neurotoxic, inducing paraplegia in naive rats when administered intrathecally, increasing SCI dysfunction in rodents, and increasing symptoms of pain [12, 27, 49, 65–68]. Dynorphin A, the endogenous ligand of KOR, is upregulated after nerve injury, in response to pain signaling. However, while neuronal synthesis of Dynorphin is linked to analgesia, glia-mediated production of Dynorphin A is associated with the development of allodynia [69, 70]. It is also possible, therefore, that overproduction of dynorphin is a contributing factor to the early signs of hyperalgesia (lower vocalization threshold to mechanical stimulation) that we observed in morphine-treated rats. We believe that the increase in dynorphin concentration that we see in microglia/macrophages with morphine administration is equivalent to supra-physiological levels, that are neurotoxic after the lesion, in a rat. However, based on the results of this study we cannot assert whether the dynorphin levels we see are sufficient to induce onset of pain or undermine locomotor recovery. We recognize this as a limitation for our study and an area of interest for future research.

In addition to increasing the endogenous ligand of the KOR, the expression of KORs per se were also increased across both CD86+ and CD206+ microglia/macrophages. We posit that activation of opioid receptors on immune cells also plays an important role in the morphine-induced deficits seen after SCI. In the naïve SCI condition CD206+ cells may be beneficial, but when morphine is superimposed onto the injury it may trigger the production of Dynorphin A via the activation of opioid receptors. Indeed, in neurons, morphine activation of ORs initiates the ERK/MAPK pathway downstream from β-arrestin signaling that results in upregulation of pro-dynorphin [48]. Using Western blot analysis, we assessed changes in the concentration of proteins downstream of the inflammatory β-arrestin pathway in ex vivo CD11b+ cells, isolated with immune-magnetic cell sorting. We found that, in addition to dynorphin and pro-dynorphin, both β-arrestin and ERK1 protein levels are elevated 30-min after morphine administration. These data suggest that, as seen in neurons, activation of opioid receptors on immune cells also engages the β-arrestin pathway. However, we did not see this effect when we delayed tissue collection by 24-h post-administration, suggesting that effects at the early timepoint are due to active binding of morphine rather than permanent signaling changes induced by morphine. Further, when we quantified protein levels 24-h post administration in the 7-day morphine group, we saw a reduction of pro-dynorphin levels but not of dynorphin compared to saline-treated contused animals. This might reflect a depletion of this precursor protein as a result of the increased synthesis and release of dynorphin induced by morphine. Therefore, while we had initially hypothesized that the adverse effects of morphine were due to direct binding to KORs on microglia and macrophages, it is possible that morphine binds to any opioid receptors on immune cells, including CD206+ microglia and macrophages that are generally beneficial for the resolution of injury, to increase their production of dynorphin and exacerbate the injury.

We did not assess whether morphine increases the expression of other opioid receptor subtypes, but extensive literature shows that morphine will simultaneously bind to KORs as well as to other ORs on glia to increase the immune response [18, 71]. It is also important to note that we had to restrict the number of antibodies we probed within our flow panels, to prevent fluorescence spillover. Thus, a limitation of this study is also the lack of a neutrophil-specific marker, like Ly6G, that would allow us to differentiate between these cells and macrophages, as both populations are CD11b+/CD45^High^. Similarly, we were limited in the number of pro- and anti-inflammatory glial markers we were able to probe within each set. It would have also been informative to conduct a longer assessment of behavior and recovery beyond 7 days post-injury. While this has been partially addressed in previous studies from our laboratory [8, 13, 15], this is a limitation of the present study and must be incorporated in future studies to strengthen the implications of our research. Additionally, in this study we focused exclusively on male rats. It is important that future studies explore these effects in female populations as there might be important sex-differences in opioid-immune interactions after SCI.

Overall, however, based on the data presented in this study, as well as extensive literature, we posit that morphine binds and activates ORs that are found on microglia/macrophages and changes both their expression and function. This in turn induces the production of neurotoxic factors that magnify the secondary injury cascade. Immediately after an SCI there is a chain of immune and inflammatory signals that is triggered in response to the cellular damage from the injury. These molecular signals activate immuno-competent cells and recruit peripheral immune cells to infiltrate the injury. Administration of morphine immediately after the injury seems to further increase the proliferation and activation of these cells at the injury.

After an injury, microglia and macrophages are typically recruited to contain inflammatory material and help with tissue repair, as well as to perform removal of debris cause by the injury. However, an excessive response by these immune cells will exacerbate the injury and impairs recovery in rodent models of SCI [57, 72, 73]. We propose that morphine induces these deficits by increasing the expression of opioid receptors and activating them to modify the innate response to the injury. We posit that morphine is acting through these opioid receptors to activate proteins related to the β-arrestin pathway, including ERK 1/2 and p38 MAPK, which are known to activate inflammatory and apoptotic signals. Additionally, MAPK/ERK pathways control CREB production of dynorphin. Based on extensive literature, we propose that the supra-physiological levels of dynorphin that are being synthesized by microglia/macrophages contribute to the heightened neuronal death we see in our contusion model by augmenting the vulnerability of neurons already stressed by the injury. Histological studies from our laboratory have shown that morphine administration after a contusion injury increases the relative size of the lesion. It also dramatically decreases the expression of the neuronal marker NeuN+ and increased the Fluoro-Jade C (FJC) expression—a marker for apoptotic mature neurons—to the point where there were essentially no NeuN+ cells were left across the extent of the lesion [14], suggesting that morphine administration after injury exacerbates cell death at the site of lesion. We believe that activation of opioid receptors on microglia and macrophages fundamentally changes the function of this cells and causes a neurotoxic environment around the lesion. If this is the case, blocking microglial activation or KORs during i.v. morphine administration should protect recovery of function, as we have seen in the intrathecal morphine administration model [13]. Use of KOR antagonists as adjuvants to morphine might protect recovery of function, while affording effective pain relief in the acute phase of SCI. By identifying the critical molecular changes that underlie the negative effects of morphine after an SCI, we aim to improve the safety of opioids while maintaining their powerful analgesic properties to effectively treat pain after SCI.

## Conclusion

In summary, we found that intravenous morphine administration after SCI increases the total number of activated microglia and macrophages at the site of injury, including CD86 + and CD206 + cells, while simultaneously triggering the β-arrestin pathway and the production of dynorphin in these cells. Therefore, whereas in the saline-treated SCI condition an increase in CD206 + macrophages might be beneficial for injury resolution, activation of these cells with morphine after SCI increases the concentration of dynorphin. Previous studies have shown that supraphysiological quantities of dynorphin after SCI are neurotoxic. Increased dynorphin synthesis likely contributes to the impairment of locomotor recovery and increase in cell death we have observed in our rodent contusion model.

## Supplementary Information

**Additional file 1: Figure S1.** Quantification of % live cells in heterogenous mixture of disassociated cells collected from the site of injury across treatment groups using two different live/dead staining methods 1) Countess (Life Technologies, Carlsbad, CA, USA), and 2) FlowJo analysis of Zombie NI R (live/dead stain) used in flow cytometry protocol.

**Additional file 2: Figure S2.** Quantification of microglia and macrophages in CD206 set using flow cytometry. The contusion injury significantly increases the number of CD11b total positive cells (A), percentage of CD11 b positive cells (B), total number of macrophages (C), and total number of microglia (D) at the site of injury relative to a sham surgery. After 3 days of morphine administration, contused animals also had a significantly higher total number of CD11b positive cells (A), percentage of CD11b positive cells (B), total number of macrophages (C), and total number of microglia (D) compared with vehicle SCI controls. There was no significant effect of treatment with 1 or 7 days of morphine administration. Results shown as Mean S.E.M. *p < 0.05, n = 5–6.

**Additional file 3: Figure S3.** Western blot full gel image for protein extracted from animals that received 1 day of drug administration.

**Additional file 4: Figure S4. **Western blot full gel image for protein extracted from animals that received 7 days of drug administration.

## Data Availability

The datasets used and/or analyzed during the current study are available from the corresponding author on reasonable request.

## Abbreviations

ANOVA: Analysis of variance
BBB Scale: Basso, Beattie and Bresnahan locomotor scale
BSA: Bovine Serum Albumin
CD11B: Cluster of Differentiation Molecule 11B
CD206: Cluster of Differentiation Molecule 206
CD45: Cluster of Differentiation Molecule 45
CD86: Cluster of Differentiation Molecule 86
CREB: CAMP response element-binding protein
ERK: Extracellular signal-regulated kinases
FACS: Fluorescence-activated cell sorting
FITC: Fluorescein isothiocyanate
HBSS: Hanks' Balanced Salt Solution
i.t.: Intrathecal
i.v.: Intravenous
KOR: Kappa opioid receptor
MACS: Magnetic-activated cell sorting
MAPK: Mitogen-activated protein kinase
NIR: Near-infrared
nor-BNI: Norbinaltorphimine
ORs: Opioid receptors
PBS: Phosphate-buffered saline
PE: Phycoerythrin
PVDF: Polyvinylidene fluoride
RIPA: Radioimmunoprecipitation assay buffer
S.E.M.: Standard error of the mean
SCI: Spinal cord injury
SDS-PAGE: Sodium dodecyl sulphate–polyacrylamide gel electrophoresis
TBST: Tris-buffered saline with 0.1% Tween® 20 Detergent

## References

[1] Siddall PJ (1999). Pain report and the relationship of pain to physical factors in the first 6 months following spinal cord injury. Pain.

[2] Siddall PJ (2003). A longitudinal study of the prevalence and characteristics of pain in the first 5 years following spinal cord injury. Pain.

[3] Katz J, Seltzer Z (2009). Transition from acute to chronic postsurgical pain: risk factors and protective factors. Expert Rev Neurother.

[4] Kyranou M, Puntillo K (2012). The transition from acute to chronic pain: might intensive care unit patients be at risk?. Ann Intensive Care.

[5] Pergolizzi JV, Raffa RB, Taylor R (2014). Treating acute pain in light of the chronification of pain. Pain Manag Nurs.

[6] Stampas A (2020). The first 24 h: opioid administration in people with spinal cord injury and neurologic recovery. Spinal Cord.

[7] Hook MA (2007). The impact of morphine after a spinal cord injury. Behav Brain Res.

[8] Hook MA (2009). Intrathecal morphine attenuates recovery of function after a spinal cord injury. J Neurotrauma.

[9] Hook MA (2011). An IL-1 receptor antagonist blocks a morphine-induced attenuation of locomotor recovery after spinal cord injury. Brain Behav Immun.

[10] Woller SA (2012). Analgesia or addiction?: implications for morphine use after spinal cord injury. J Neurotrauma.

[11] Woller SA (2014). Morphine self-administration following spinal cord injury. J Neurotrauma.

[12] Aceves M, Mathai BB, Hook MA (2016). Evaluation of the effects of specific opioid receptor agonists in a rodent model of spinal cord injury. Spinal Cord.

[13] Aceves M (2017). Nor-binaltorphimine blocks the adverse effects of morphine after spinal cord injury. J Neurotrauma.

[14] Hook MA (2017). Neurobiological effects of morphine after spinal cord injury. J Neurotrauma.

[15] Aceves M (2019). Morphine increases macrophages at the lesion site following spinal cord injury: protective effects of minocycline. Brain Behav Immun.

[16] Faulkner JR (2004). Reactive astrocytes protect tissue and preserve function after spinal cord injury. J Neurosci.

[17] Probert L (2000). TNFR1 signalling is critical for the development of demyelination and the limitation of T-cell responses during immune-mediated CNS disease. Brain.

[18] Hutchinson MR (2011). Exploring the neuroimmunopharmacology of opioids: an integrative review of mechanisms of central immune signaling and their implications for opioid analgesia. Pharmacol Rev.

[19] Bunn SJ, Hanley MR, Wilkin GP (1985). Evidence for a kappa-opioid receptor on pituitary astrocytes: an autoradiographic study. Neurosci Lett.

[20] Eriksson PS (1993). Kappa-opioid receptors on astrocytes stimulate L-type Ca2+ channels. Neuroscience.

[21] Belcheva MM (2005). Mu and kappa opioid receptors activate ERK/MAPK via different protein kinase C isoforms and secondary messengers in astrocytes. J Biol Chem.

[22] Bruchas MR (2006). Kappa opioid receptor activation of p38 MAPK is GRK3- and arrestin-dependent in neurons and astrocytes. J Biol Chem.

[23] Bruchas MR, Chavkin C (2010). Kinase cascades and ligand-directed signaling at the kappa opioid receptor. Psychopharmacology.

[24] Bruchas MR (2007). Stress-induced p38 mitogen-activated protein kinase activation mediates kappa-opioid-dependent dysphoria. J Neurosci.

[25] Xu M (2007). Sciatic nerve ligation-induced proliferation of spinal cord astrocytes is mediated by kappa opioid activation of p38 mitogen-activated protein kinase. J Neurosci.

[26] Kreibich AS, Blendy JA (2004). cAMP response element-binding protein is required for stress but not cocaine-induced reinstatement. J Neurosci.

[27] Faden AI (1990). Opioid and nonopioid mechanisms may contribute to dynorphin's pathophysiological actions in spinal cord injury. Ann Neurol.

[28] Jain NB (2015). Traumatic spinal cord injury in the United States, 1993–2012. JAMA.

[29] Rodriguez-Meza MV (2016). Clinical and demographic profile of traumatic spinal cord injury: a Mexican hospital-based study. Spinal Cord.

[30] Brakel K (2021). Inflammation increases the development of depression behaviors in male rats after spinal cord injury. Brain Behav Immun Health.

[31] Grau JW (2004). Uncontrollable stimulation undermines recovery after spinal cord injury. J Neurotrauma.

[32] Sedy J (2008). Methods for behavioral testing of spinal cord injured rats. Neurosci Biobehav Rev.

[33] D'amour FE, Smith DL (1941). A method for determining loss of pain sensation. J Pharmacol Exp Ther.

[34] Jeffrey SM, Sonya GW, You W (2001). Assessing nociception in murine subjects. Methods in pain research.

[35] Basso DM, Beattie MS, Bresnahan JC (1995). A sensitive and reliable locomotor rating scale for open field testing in rats. J Neurotrauma.

[36] Ferguson AR (2004). A simple post hoc transformation that improves the metric properties of the BBB scale for rats with moderate to severe spinal cord injury. J Neurotrauma.

[37] Springer T (1979). Mac-1: a macrophage differentiation antigen identified by monoclonal antibody. Eur J Immunol.

[38] Kantor AB (1992). Differential development of progenitor activity for three B-cell lineages. Proc Natl Acad Sci USA.

[39] McFarland HI (1992). CD11b (Mac-1): a marker for CD8+ cytotoxic T cell activation and memory in virus infection. J Immunol.

[40] Vremec D (1992). The surface phenotype of dendritic cells purified from mouse thymus and spleen: investigation of the CD8 expression by a subpopulation of dendritic cells. J Exp Med.

[41] Sedgwick JD (1991). Isolation and direct characterization of resident microglial cells from the normal and inflamed central nervous system. Proc Natl Acad Sci USA.

[42] Ford AL (1995). Normal adult ramified microglia separated from other central nervous system macrophages by flow cytometric sorting. Phenotypic differences defined and direct ex vivo antigen presentation to myelin basic protein-reactive CD4+ T cells compared. J Immunol.

[43] Badie B, Schartner JM (2000). Flow cytometric characterization of tumor-associated macrophages in experimental gliomas. Neurosurgery.

[44] Martin E (2017). Analysis of microglia and monocyte-derived macrophages from the central nervous system by flow cytometry. J Vis Exp.

[45] Yao A (2014). Programmed death 1 deficiency induces the polarization of macrophages/microglia to the M1 phenotype after spinal cord injury in mice. Neurotherapeutics.

[46] Francos-Quijorna I (2016). IL-4 drives microglia and macrophages toward a phenotype conducive for tissue repair and functional recovery after spinal cord injury. Glia.

[47] Kobashi S (2020). Transplantation of M2-deviated microglia promotes recovery of motor function after spinal cord injury in mice. Mol Ther.

[48] Carlezon WA, Duman RS, Nestler EJ (2005). The many faces of CREB. Trends Neurosci.

[49] Long JB (1988). Neurological dysfunction after intrathecal injection of dynorphin A (1–13) in the rat. II. Nonopioid mechanisms mediate loss of motor, sensory and autonomic function. J Pharmacol Exp Ther.

[50] Bomstein Y (2003). Features of skin-coincubated macrophages that promote recovery from spinal cord injury. J Neuroimmunol.

[51] Ma SF (2015). Adoptive transfer of M2 macrophages promotes locomotor recovery in adult rats after spinal cord injury. Brain Behav Immun.

[52] Gensel JC (2017). Predictive screening of M1 and M2 macrophages reveals the immunomodulatory effectiveness of post spinal cord injury azithromycin treatment. Sci Rep.

[53] Jeong SJ (2017). Intravenous immune-modifying nanoparticles as a therapy for spinal cord injury in mice. Neurobiol Dis.

[54] Hu G (2018). Astrocyte EV-induced lincRNA-Cox2 regulates microglial phagocytosis: implications for morphine-mediated neurodegeneration. Mol Ther Nucleic Acids.

[55] Godai K (2014). Peripheral administration of morphine attenuates postincisional pain by regulating macrophage polarization through COX-2-dependent pathway. Mol Pain.

[56] Kigerl KA (2009). Identification of two distinct macrophage subsets with divergent effects causing either neurotoxicity or regeneration in the injured mouse spinal cord. J Neurosci.

[57] David S, Kroner A (2011). Repertoire of microglial and macrophage responses after spinal cord injury. Nat Rev Neurosci.

[58] Hakim R (2021). Spinal cord injury induces permanent reprogramming of microglia into a disease-associated state which contributes to functional recovery. J Neurosci.

[59] Poulen G (2021). Inhibiting microglia proliferation after spinal cord injury improves recovery in mice and nonhuman primates. Theranostics.

[60] Pannell M (2016). Adoptive transfer of M2 macrophages reduces neuropathic pain via opioid peptides. J Neuroinflamm.

[61] Przewlocki R, Shearman GT, Herz A (1983). Mixed opioid/nonopioid effects of dynorphin and dynorphin related peptides after their intrathecal injection in rats. Neuropeptides.

[62] Cox BM (1985). Effects of traumatic injury on dynorphin immunoreactivity in spinal cord. Neuropeptides.

[63] Faden AI (1985). Increased dynorphin immunoreactivity in spinal cord after traumatic injury. Regul Pept.

[64] Mika J, Obara I, Przewlocka B (2011). The role of nociceptin and dynorphin in chronic pain: implications of neuro-glial interaction. Neuropeptides.

[65] Hu WH (1996). Dynorphin neurotoxicity induced nitric oxide synthase expression in ventral horn cells of rat spinal cord. Neurosci Lett.

[66] Laughlin TM (2000). Cytokine involvement in dynorphin-induced allodynia. Pain.

[67] Adjan VV (2007). Caspase-3 activity is reduced after spinal cord injury in mice lacking dynorphin: differential effects on glia and neurons. Neuroscience.

[68] Mika J (2010). Minocycline reduces the injury-induced expression of prodynorphin and pronociceptin in the dorsal root ganglion in a rat model of neuropathic pain. Neuroscience.

[69] Xu M (2004). Neuropathic pain activates the endogenous kappa opioid system in mouse spinal cord and induces opioid receptor tolerance. J Neurosci.

[70] Zhu X (2006). Spinal cord dynorphin expression increases, but does not drive microglial prostaglandin production or mechanical hypersensitivity after incisional surgery in rats. Pain.

[71] Tegeder I, Geisslinger G (2004). Opioids as modulators of cell death and survival–unraveling mechanisms and revealing new indications. Pharmacol Rev.

[72] Bellon T (2011). Alternative activation of macrophages in human peritoneum: implications for peritoneal fibrosis. Nephrol Dial Transplant.

[73] Brown GC, Neher JJ (2012). Eaten alive! Cell death by primary phagocytosis: 'phagoptosis'. Trends Biochem Sci.

