# Supplementary material for: Morphine-induced changes in the function of microglia and macrophages after acute spinal cord injury
Source: BMC Neurosci. 2022 Oct 10;23:58. doi: 10.1186/s12868-022-00739-3 (PMC9552511; doi:10.1186/s12868-022-00739-3)
Supplement: Supplementary file 1 — Additional file 1: Figure S1. Quantification of % live cells in heterogenous mixture of disassociated cells collected from the site of injury across treatment groups using two different live/dead staining methods 1) Countess (Life Technologies, Carlsbad, CA, USA), and 2) FlowJo analysis of Zombie NI R (live/dead stain) used in flow cytometry protocol. [file 12868_2022_739_MOESM1_ESM.pdf]

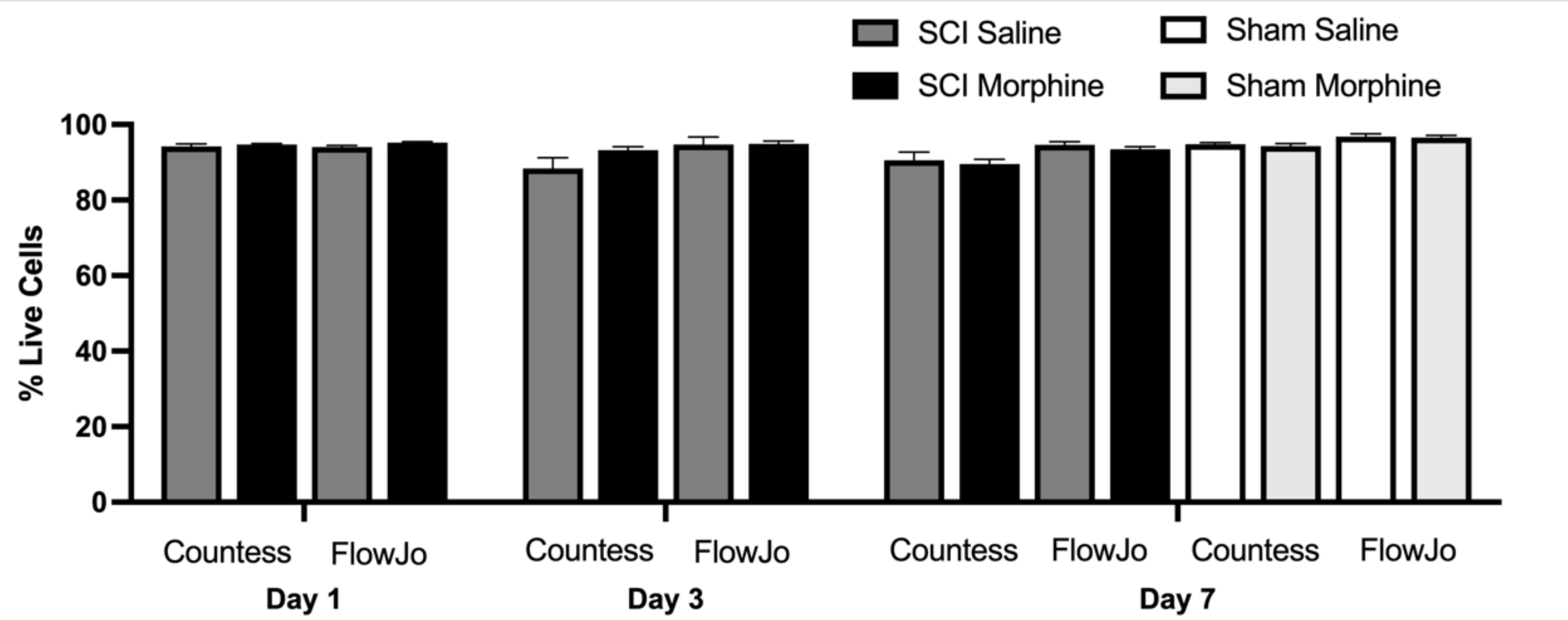

Additional file 1. Quantification of % live cells in heterogenous mixture of disassociated cells collected from the site of injury across treatment groups using two different live/dead staining methods 1) Countess (Life Technologies, Carlsbad, CA, USA), and 2) FlowJo analysis of Zombie NIR (live/dead stain) used in flow cytometry protocol.
