# Supplementary material for: Morphine-induced changes in the function of microglia and macrophages after acute spinal cord injury
Source: BMC Neurosci. 2022 Oct 10;23:58. doi: 10.1186/s12868-022-00739-3 (PMC9552511; doi:10.1186/s12868-022-00739-3)
Supplement: Supplementary file 2 — Additional file 2: Figure S2. Quantification of microglia and macrophages in CD206 set using flow cytometry. The contusion injury significantly increases the number of CD11b total positive cells (A), percentage of CD11 b positive cells (B), total number of macrophages (C), and total number of microglia (D) at the site of injury relative to a sham surgery. After 3 days of morphine administration, contused animals also had a significantly higher total number of CD11b positive cells (A), percentage of CD11b positive cells (B), total number of macrophages (C), and total number of microglia (D) compared with vehicle SCI controls. There was no significant effect of treatment with 1 or 7 days of morphine administration. Results shown as Mean S.E.M. *p < 0.05, n = 5–6. [file 12868_2022_739_MOESM2_ESM.pdf]

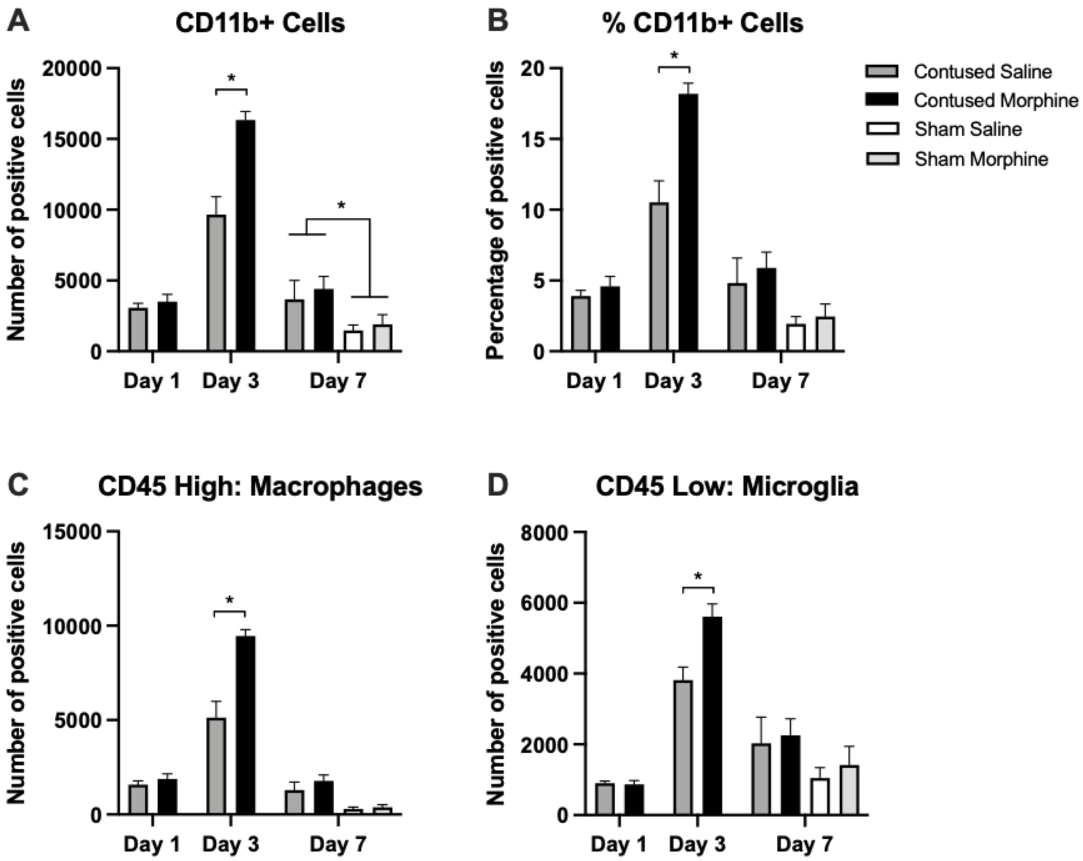

Additional Figure 2. Quantification of microglia and macrophages in CD206 set using flow cytometry. The contusion injury significantly increases the number of CD11b total positive cells (A), percentage of CD11b positive cells (B), total number of macrophages (C), and total number of microglia (D) at the site of injury relative to a sham surgery. After 3 days of morphine administration, contused animals also had a significantly higher total number of CD11b positive cells (A), percentage of CD11b positive cells (B), total number of macrophages (C), and total number of microglia (D) compared with vehicle SCI controls. There was no significant effect of treatment with 1 or 7 days of morphine administration. Results shown as Mean ± S.E.M. $*p < 0.05$, n=5-6.
