# Supplementary material for: Morphine-induced changes in the function of microglia and macrophages after acute spinal cord injury
Source: BMC Neurosci. 2022 Oct 10;23:58. doi: 10.1186/s12868-022-00739-3 (PMC9552511; doi:10.1186/s12868-022-00739-3)
Supplement: Supplementary file 4 — Additional file 4: Figure S4. Western blot full gel image for protein extracted from animals that received 7 days of drug administration. [file 12868_2022_739_MOESM4_ESM.pdf]

7 Days of Administration

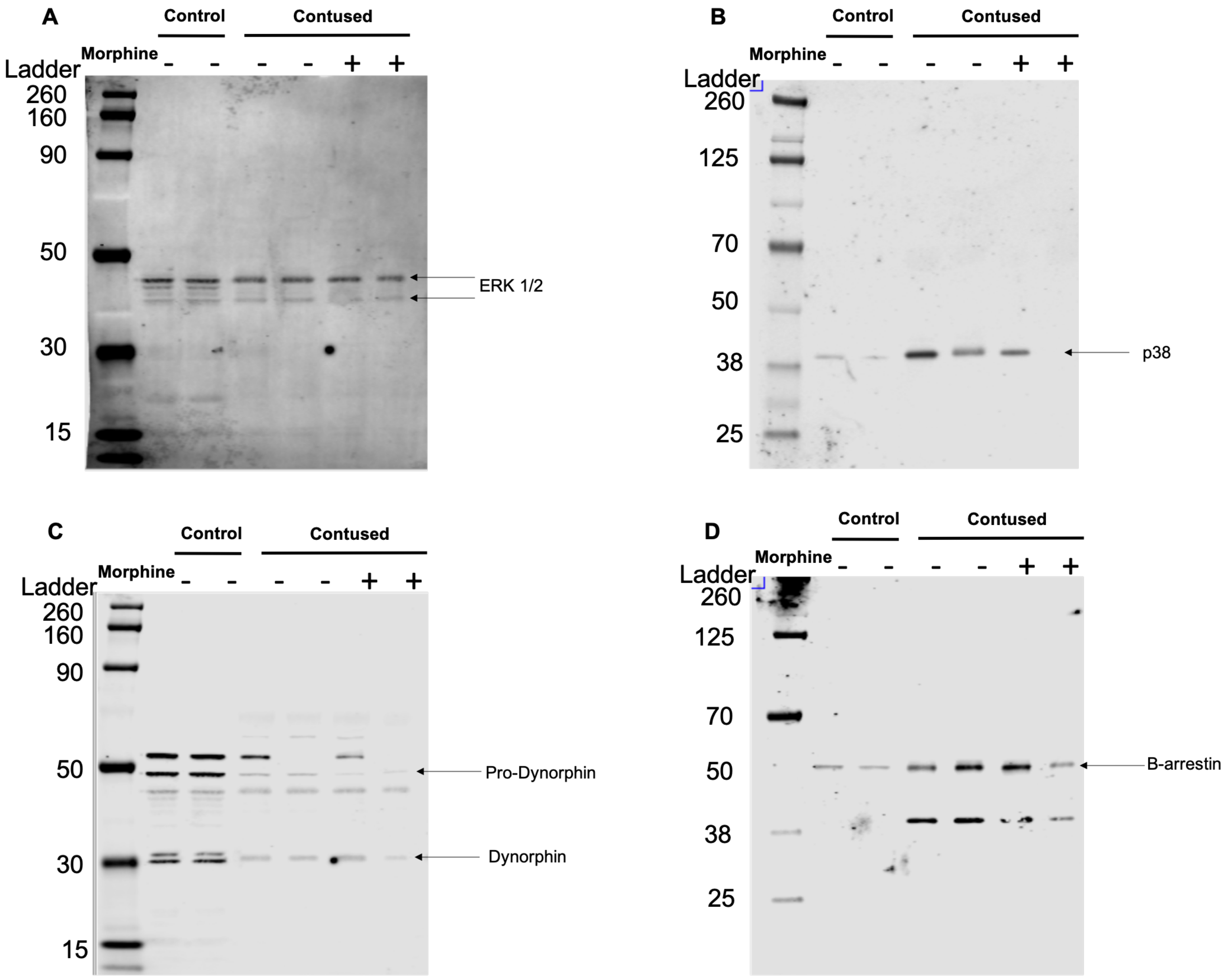

Additional Figure 4. Western blot full gel image for protein extracted from animals that received 7 days of drug administration.
