# Supplementary material for: Morphine-induced changes in the function of microglia and macrophages after acute spinal cord injury
Source: BMC Neurosci. 2022 Oct 10;23:58. doi: 10.1186/s12868-022-00739-3 (PMC9552511; doi:10.1186/s12868-022-00739-3)
Supplement: Supplementary file 3 — Additional file 3: Figure S3. Western blot full gel image for protein extracted from animals that received 1 day of drug administration. [file 12868_2022_739_MOESM3_ESM.pdf]

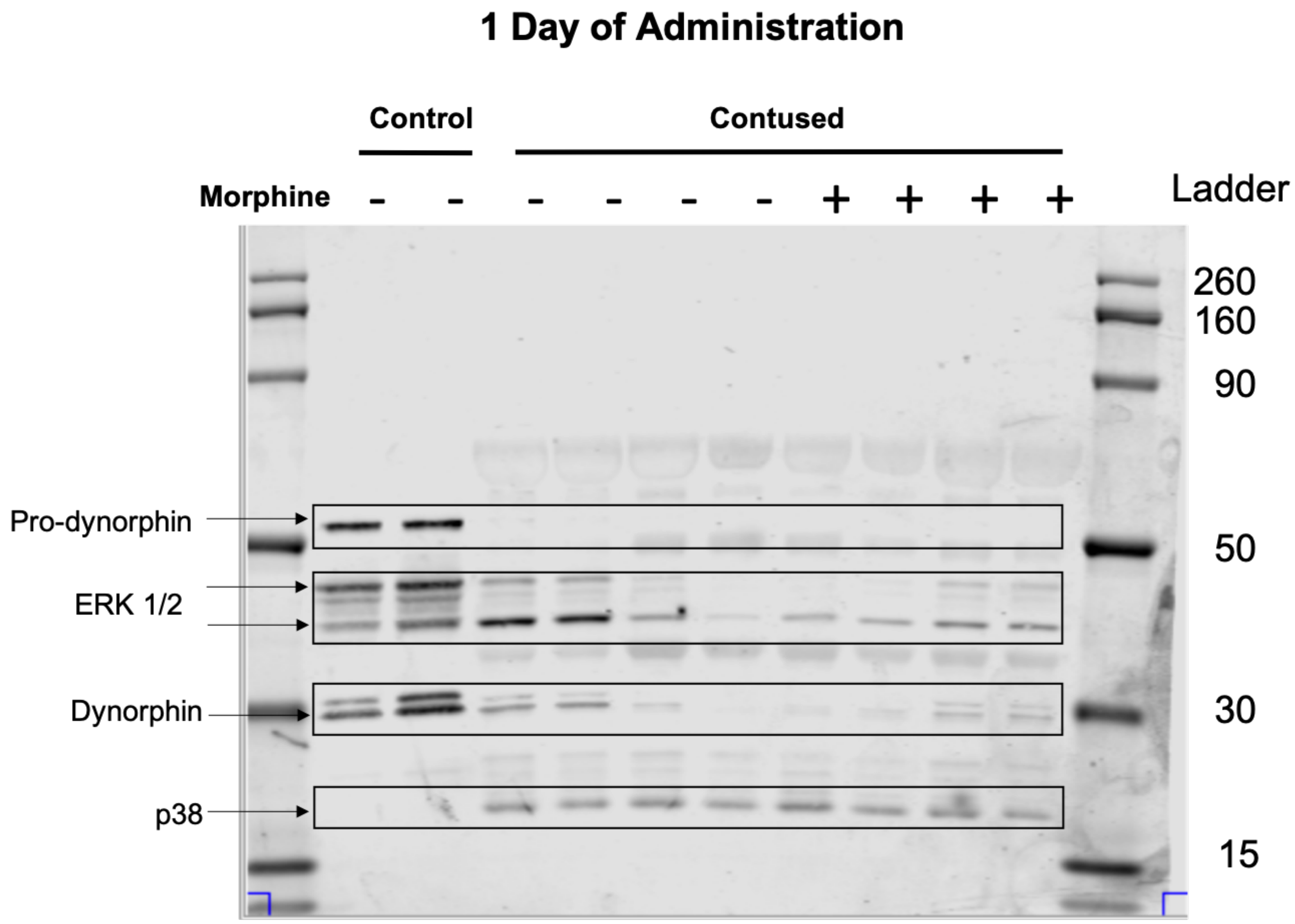

Additional Figure 3. Western blot full gel image for protein extracted from animals that received 1 day of drug administration
